# A versatile jellyfish-like robotic platform for effective underwater propulsion and manipulation

**DOI:** 10.1126/sciadv.adg0292

**Published:** 2023-04-12

**Authors:** Tianlu Wang, Hyeong-Joon Joo, Shanyuan Song, Wenqi Hu, Christoph Keplinger, Metin Sitti

**Affiliations:** ^1^ Physical Intelligence Department, Max Planck Institute for Intelligent Systems, Stuttgart 70569, Germany.; ^2^ Department of Information Technology and Electrical Engineering, ETH Zurich, Zurich 8092, Switzerland.; ^3^ Robotic Materials Department, Max Planck Institute for Intelligent Systems, Stuttgart 70569, Germany.; ^4^ Bioinspired Autonomous Miniature Robots Group, Stuttgart 70569, Germany.; ^5^ Paul M. Rady Department of Mechanical Engineering, University of Colorado Boulder, Boulder, CO 80309, USA.; ^6^ Materials Science and Engineering Program, University of Colorado Boulder, Boulder, CO 80309, USA.; ^7^ School of Medicine and College of Engineering, Koç University, Istanbul 34450, Turkey.

## Abstract

Underwater devices are critical for environmental applications. However, existing prototypes typically use bulky, noisy actuators and limited configurations. Consequently, they struggle to ensure noise-free and gentle interactions with underwater species when realizing practical functions. Therefore, we developed a jellyfish-like robotic platform enabled by a synergy of electrohydraulic actuators and a hybrid structure of rigid and soft components. Our 16-cm-diameter noise-free prototype could control the fluid flow to propel while manipulating objects to be kept beneath its body without physical contact, thereby enabling safer interactions. Its against-gravity speed was up to 6.1 cm/s, substantially quicker than other examples in literature, while only requiring a low input power of around 100 mW. Moreover, using the platform, we demonstrated contact-based object manipulation, fluidic mixing, shape adaptation, steering, wireless swimming, and cooperation of two to three robots. This study introduces a versatile jellyfish-like robotic platform with a wide range of functions for diverse applications.

## Teaser

Electrohydraulic actuation of a hybrid architecture enables noise-free, fast, energy-efficient propulsion and diverse functions.

## INTRODUCTION

Waterbodies cover more than 70% of the earth’s surface, but over 80% have not been mapped, observed, or explored. In particular, there have been few scientific investigations into the benthic habitat. Such studies are urgent regarding environmental protection, especially given humans’ increasingly negative impact on waterbodies in recent years. For example, the tropical coral reef, a great biodiversity source, occupies only 0.1% of the ocean, but it serves as the habitat for 25% of all known marine species (although many more species are yet to be discovered). At the same time, 70% of marine litter is estimated to sink to the seabed. Plastics compose more than 60% of this litter, taking hundreds of years to degrade in the ocean. They would inevitably cause health problems for human beings through the global carbon cycle and need to be appropriately investigated and recycled to avoid irreversible environmental damage (*1*). Therefore, there is an urgent need to conduct against-gravity and large-scale underwater object manipulation tasks, i.e., we must sample organisms and litter and transport them upward. Since these tasks are labor intensive, underwater robots could assist and even replace human beings (*2*).

To this end, these delicate machines should be able to manipulate objects in a fast but energy-efficient manner. They must operate in a gentle and noise-free manner to safely interact with underwater species without causing deleterious disruptions (*2*). Most of the current underwater robot designs can be divided into two subtypes: zoomorphic types and unmanned vehicle types, although some works have also been inspired by other natural morphologies, such as plants (*3*). The research on the zoomorphic types includes fish-like undulatory swimmers (*4*–*7*), octopus-like robots (*8*), jellyfish-like robots (*9*), and other morphologies (*10*). Most studies focus on body-undulatory swimmers and explore different topics, from basic biomechanics studies (*5*) to real-world applications (*11*). Although recent works have systematically investigated ways to improve the energy efficiency of propulsion, e.g., by tuning the body stiffness distributions (*4*, *5*), these bioinspired undulatory platforms have not shown important functions other than underwater inspection, limiting their possible applications. Moreover, the most commonly used actuation methods are either electrical motor–based transmission mechanisms (*5*) or hydraulic pumps (*11*) in outdoor field operations, both of which make noise and vibration. On the other hand, unmanned vehicles, including commercialized ones such as REMUS 100-S, mostly rely on electrical motors and are primarily designed to inspect and collect data. Furthermore, unmanned vehicles are rigid and bulky, and their design has not been optimized for exploring and sampling in complex and unstructured environments such as coral reefs. In summary, existing prototypes cannot interact with aquatic species gently in a noise-free manner while accomplishing diverse tasks.

In nature, jellyfish are one of the most energy-efficient underwater animals due to their locomotion modes, i.e., jet-based or paddling-based swimming (*9*). Moreover, how jellyfish swim combines effective fluidic propulsion and object manipulation, which is beneficial for predation (*8*, *12*, *13*). Furthermore, their deformable soft body structure allows them to adapt to and navigate unstructured environments. These properties make jellyfish one of the most popular animal models to guide the design of a new generation of underwater vehicles (*9*). Several robotic prototypes have already been inspired by jellyfish and developed using various actuation techniques, such as electrical motors (*14*), hydraulic pumps (*15*), pneumatic pumps (*16*), shape memory alloys (SMAs) (*17*–*19*), dielectric elastic actuators (DEAs) (*20*, *21*), and ionic polymer metal composite actuators (*22*). However, electric motors and hydraulic pumps require bulky transmission mechanisms, which inevitably make noise and vibration; SMAs consume a lot of power when heated up. Although DEA-actuated robots have enabled fast propulsion in the air and on the ground (*23*, *24*), they struggle to provide quick underwater propulsion since the demonstrated prototypes could only generate small strokes (*20*, *21*, *25*). In addition, these robotic prototypes in literature lack the capability to manipulate objects.

**Fig. 1.**
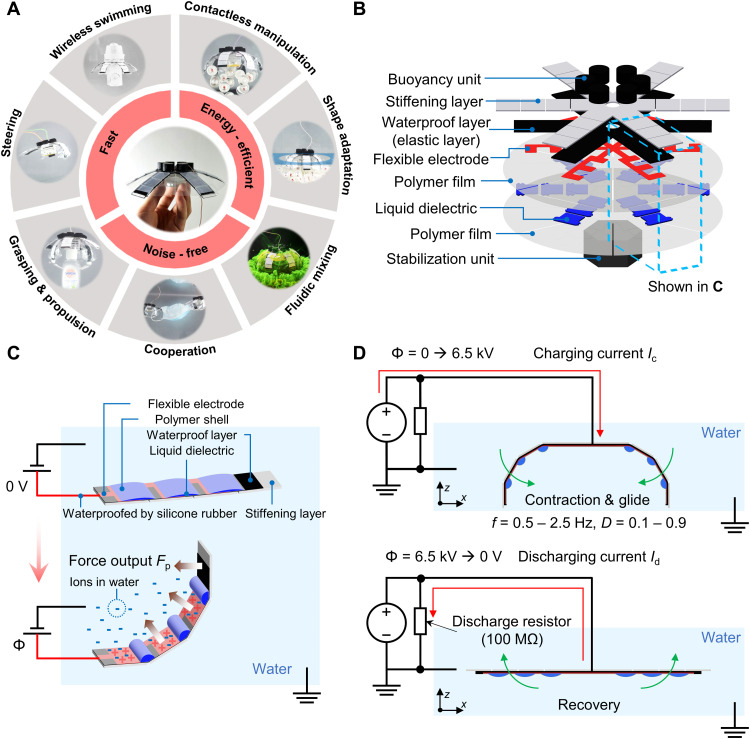
Robot design and the principle of actuation. (**A**) Key features of the HASEL jellyfish robot platform. (**B**) Two-dimensional (2D) layered design of the robot. (**C**) Details of a lappet (leg) design and the principle of electrohydraulic actuation to generate large deformation and force output. When a high voltage (HV) Φ is applied, the Maxwell stress between the flexible electrode and the grounded water causes the polymer shell to zip. It also displaces the liquid dielectric, causing the joints to bend with the given geometric constraints. Thus, the bending movement of the rigid stiffening layer generates large paddling forces for propulsion. The actuation is identical for the three interconnected electrodes in the three joints. The paddling force of the optimized joint design *F*_p_max_ was measured to be 54.8 mN (see Fig. 2B). (**D**) Generation of jellyfish-like locomotion. The charging period corresponds to the contraction and glide phase of jellyfish locomotion, and the discharging period corresponds to the recovery phase. We designed customized compact driving electronics to generate a cyclic HV Φ (Φ_max_ = 6.5 kV) for the charging current *I*_c_ and discharging current *I*_d_. The 100-megohm resistor enables the discharge of the actuators when Φ = 0 V.

To overcome these challenges, we have developed a new robotic jellyfish-like platform, termed the HASEL jellyfish robot, which is comparable in size to an adult *Aurelia aurita* (i.e., the moon jellyfish). This platform is actuated by soft actuators (*26*, *27*), namely, hydraulically amplified self-healing electrostatic (HASEL) actuators (*28*–*31*), which combine the advantages of DEAs and hydraulic actuators, thereby achieving versatile and high-performance actuation. Enabled by a synergetic combination of these electrohydraulic actuators and a hybrid structure comprising both rigid and soft components, our underwater jellyfish-like robot realized fast and noise-free propulsion, with against-gravity speed up to 6.1 cm/s, while only consuming power around 100 mW. Moreover, given the design and the individual lappet controllability, the robots introduced here fulfilled diverse underwater functions, such as contact-based and contactless object manipulation, fluid mixing, shape adaptation, and steering, without using any bulky or rigid transmission mechanisms. Next, on top of a single robot design, we demonstrated that a team of these robots could further enhance their object manipulation capabilities. Last, we developed a fully wireless prototype and tested it outdoors; all the power and electronics were integrated onboard. This new noise-free robotic jellyfish platform, along with the demonstrated advances in both propulsion performance and practical functions, will help inspiring the next generation of underwater vehicles for real-world applications (Fig. 1A).

## RESULTS

### Design of the HASEL jellyfish robot

The robot was designed to have a diameter, *D*, of 160 mm so that it would be comparable in size to an adult *A. aurita* (Fig. 1A). The robot has six lappets, and each one can be controlled individually for diverse functions. Each lappet has flexible rotational joints powered by HASEL actuators (*29*). Here, by mentioning HASEL actuators, we generally refer to the respective platform technology for actuation, for which many different types of actuation geometries have been introduced (*28*, *29*, *32*). We fabricated the robot in a two-dimensional (2D) manner; we stacked the polymer shell (formed by two sealed films), the flexible carbon electrode, the waterproof layer, and the stiffening layer in a sequence (*33*). We added extra mass to the bottom of the robot so that the center of buoyancy would be above the center of mass, ensuring that the robot is passively stable (*34*). Then, we finely tuned the density of the robot to be 1.23 × 10^3^ kg/m^3^ by placing the symmetrically distributed buoyancy unit on the top for relatively neutral buoyancy (Fig. 1B; see the “Fabrication of the HASEL jellyfish robot” section in Materials and Methods). As a feasible improvement in future development, an active buoyance control unit, e.g., using thermally actuated liquid-gas phase transitions (*3*, *35*, *36*), can be incorporated into the platform to assist even more agile locomotion and various functions of the robot. For example, the buoyancy could be notably increased when the unit’s volume is enlarged by heating the low–boiling point liquid inside, which can increase the buoyancy and enhance the upward propulsion and transportation of the objects. On the other hand, the buoyancy will be decreased when the unit’s volume is shrunk by cooling the gas inside, which can be helpful for faster sinking.

For the wired version of the robot, which was used in most of the systematic investigations in this study, the high voltage (HV) Φ (Φ_max_ = 6.5 kV) was applied through a cable. The cable was connected to the carbon-printed HV electrode on the polymer shell, which encapsulated the liquid dielectric. The surrounding water was used as a conductive electrical ground (*37*, *38*). We could also fabricate the HV electrode using transparent conductive hydrogels for applications requiring visual disguising (*32*, *39*). When the HV was on, the Maxwell stress between the carbon electrode and the grounded water induced a zipping motion of the two sides of the polymer shell, displaced the dielectric liquid, and increased the pressure inside the actuator. Since one side of the shell was fixed onto two stiffening links, the increased pressure rotated the linked structure (Fig. 1C). Because of the simultaneous rotating movement of the actuators on each lappet, strong forces for paddling could be generated.

We designed a compact power and control electronics unit to actuate the robot by generating the desirable HV signals. The unit comprised four parts: a power source (power supply or battery), a microcontroller unit (MCU), a flyback circuit, and a multiplier circuit. In addition, we used a discharge resistor (*R*_d_ = 100 megohms) to discharge the actuators back to the rest state, i.e., flat configuration, during cyclic actuation (see the “Hardware and software to generate and control the HV” section in Materials and Methods) (*37*). We implemented software based on the Robot Operating System (ROS) framework to control the circuits. Charging and discharging the robot in controlled cycles resulted in jellyfish-like paddling motion patterns, including contraction, glide, and recovery durations (Fig. 1D) (*9*). Please note that the locomotion modes of jellyfish depend on their natural construction. While prolate jellyfish with elongated shapes typically generate a water jet for propulsion, oblate jellyfish with a plate shape mostly use a paddling motion. We used this paddling motion for the current study. Although the current design, in which directional joints achieve reciprocal locomotion, is suitable for effective swimming in the inertial fluidic regime, a bidirectional joint design could also be implemented for effective nonreciprocal propulsion in the low (viscous) to intermediate Reynolds number regimes (*29*).

**Fig. 2.**
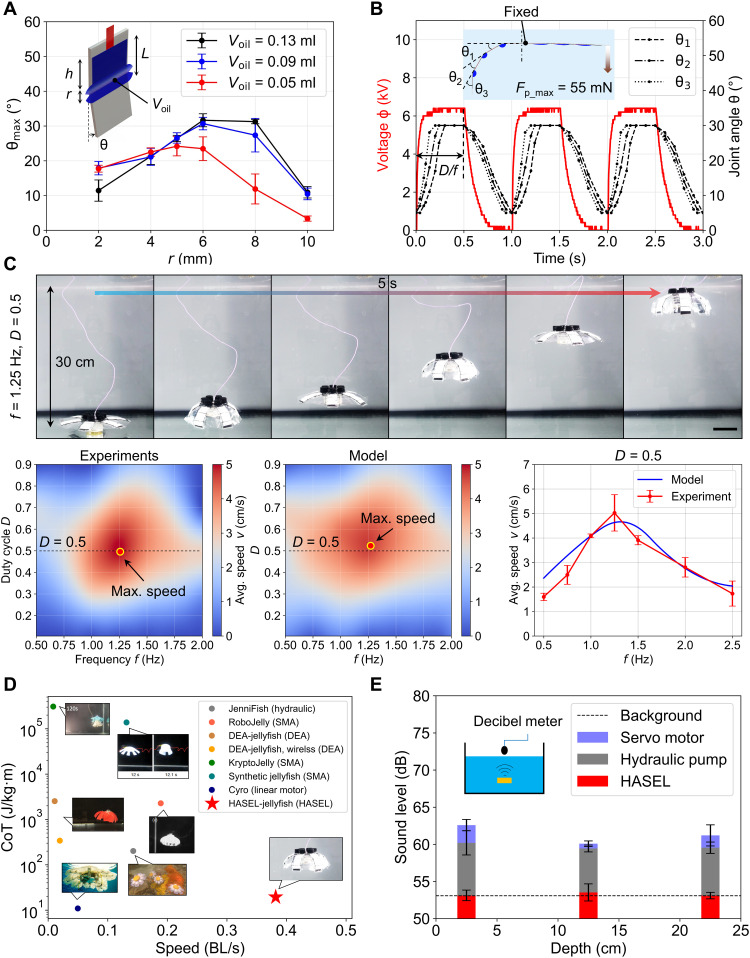
Experimental optimization of a single joint and propulsion performance. (**A**) Quasi-static experimental investigations on the effects of actuator geometry and the amount of oil *V*_oil_ on the maximum deformation angle θ_max_ for each joint. (**B**) Variations in the joint angles under the cyclic HV Φ in water (actuation frequency *f* = 1 Hz, duty cycle *D* = 0.5). Various lags, relative to Φ, were caused by different drags applied to three links. The simultaneous actuation of three joints enabled the maximum normal paddling force at the end link *F*_p_max_, which was measured to be 54.8 mN at Φ_max_ = 6.5 kV. (**C**) Investigations on the average propulsion speed *v* with different *f* and *D*. The highest *v* = 6.1 cm/s was achieved at *f* = 1.25 Hz and *D* = 0.5 (movie S1). Here, the estimated average charging current *I*_c_ was around 149.5 μA, and the discharging current *I*_d_ was around 49.8 μA, which are within the safe range for human beings and underwater species in both cases (*30*, *42*). Scale bar, 5 cm. (**D**) Comparison of *v* and the CoT between the HASEL jellyfish robot and other jellyfish-like robots that have been reported in the literature (*14*, *15*, *17*–*20*). The HASEL jellyfish robot achieved the fastest propulsion *v* = 0.4 BL/s (body length per second) and had a relatively low CoT. (**E**) Sound level measurements for different actuators. All of the actuators were operated at *f* = 1 Hz. Compared to servo motors and hydraulic pumps, the HASEL jellyfish robot was noise-free such that its noise could barely be distinguished from the background. The error bars represent the SDs for the means for the *n* ≥ 5 experimental trials.

To generate a 3D jellyfish-like umbrella-shaped bell from the 2D design for effective propulsion, the overall deformation needs to achieve 90°, i.e., the body’s outer tip goes from pointing horizontally to pointing down vertically. We first experimentally optimized how much liquid dielectric (*V*_oil_) should be used and what the geometry of each actuator for a single joint should be (i.e., the length of the electrode *h* and the length of the electrode-free shell region *r*, along with the shell length *L*, where *L = h + r*) to achieve the maximum rotation angle of each joint θ_max_ (Fig. 2A). We decided on a width *w* of 15 mm so that VHB tape (which has a width of 19 mm) could be used to secure the waterproof. The investigations have revealed that θ_max_ could reach around 30° at most given that Φ_max_ = 6.5 kV. Thus, to achieve the overall deformation of 90°, three actuators were arranged on each lappet. On the basis of previous studies, we knew that θ_max_ = 30° would generate larger torques than wider angles (*29*). The experiments showed that, for all the *V*_oil_ we investigated, θ_max_ increased and then decreased when we increased *r*. The maximum θ_max_ was achieved at *r* = 6 mm for *V*_oil_ = 0.13 ml (Fig. 2A). Therefore, we used these measurements for the design parameters. When we increased *V*_oil_ further, the pouch was overfilled, so the electrode region did not completely zip, leading to a decrease in θ_max_.

Since the customized circuits used resistors to discharge, we analyzed variations in the joint angles θ_1_ to θ_3_, i.e., the joint rotation angles with respect to the previous link, over time. This allowed us to observe the dynamic performance of the HASEL actuators in the water tank (100 cm × 50 cm × 50 cm), which was filled with tap water. We also used this tank for all of the experiments, and most of the demonstrations, that are described below. Because of the dominance of the resistors for discharging (*R*_d_ = 100 megohms), compared to the resistance of the water (from kilohms to megohms), there was no notable difference in the actuation performance in tap water or salt water. The experiments indicated that the three optimized joints achieved θ_max_ = 30°, but there were various lags in their responses to Φ (Fig. 2B). This phenomenon can be explained by the distinct fluidic drag *F*_drag_ that was applied to each link. Since the overall linked structure is the longest for θ_1_, which leads to the maximum drag, θ_1_ responds slower than θ_2_ and θ_3_. The parallel actuation of three joints results in a large force output; the normal paddling force at the end link (i.e., the θ_3_ link) for a single lappet was measured to be 54.8 mN. Thus, the paddling force of six lappets was 328.8 mN at Φ_max_ = 6.5 kV.

The HASEL actuator–based jellyfish robot design is not limited to the centimeter scale. While scaling up the design could be readily achieved by using stacked architectures, scaling down will likely require using other materials and fabrication techniques, electronics development, and design of motion patterns for effective swimming. First, lamination-based 2D fabrication techniques, as used in this study, could be replaced by two-photon polymerization. For example, the polymer shell of the actuators could be directly printed by a Nanoscribe using polydimethylsiloxane (PDMS). Further studies on material optimization are needed to overcome the nonnegligible bending stiffness at small scales (*31*). Second, advanced microelectromechanical systems (MEMS) techniques could be used for fabricating the electronics even down to the microscale for onboard control (*40*). Third, both passive structure (*13*) or asynchronous actuation of joints (*40*) along each lappet could be adopted to generate the nonreciprocal motion sequences for effective propulsion in the intermediate to viscous flow regime. After completion of the abovementioned development steps, our quasi-static (*31*) and dynamic model (*41*) could be used to predict the behavior of HASEL actuators at these smaller scales, and our customized dynamic modeling of HASEL-powered jellyfish robot could assist in the prediction of the optimal actuation parameters (frequencies and duty cycles) to achieve maximum speed.

### Propulsion performance

The large and quick deformation of the linked design, which was powered by HASEL actuators, allowed the robot to propel quickly (movie S1). We systematically investigated effects of the two actuation parameters (i.e., frequency *f* and duty cycle *D*) on average propulsion speed *v* by conducting experiments and dynamic simulations (see “Dynamics model of a swimming HASEL jellyfish robot” in the Materials and Methods). The dynamics model was beneficial for understanding the contributions of various forces (i.e., the thrust force, drag force, and acceleration reaction force) during propulsion. The results indicated that, by increasing *f* and *D*, *v* first increases and then decreases. The peak value (*v =* 6.1 cm/s) was achieved at around *f* = 1.25 Hz and *D* = 0.5 (Fig. 2C), corresponding to around 0.38 BL/s (body length per second). Therefore, the robot operates in the inertial flow regime [Reynolds number (*Re*) > 2000]. The corresponding average charging current *I*_c_ was estimated to be around 149.5 μA, while the discharging current *I*_d_ was around 49.8 μA. Thus, the current level remains within the safe range for human beings based on the safety standards provided by Underwriters Laboratories (UL) and International Electro-technical Commission (IEC) (*30*, *42*), which is around 10 mA. According to the data reported in the literature, the HASEL jellyfish robot achieved the highest speed in terms of body lengths per second among the against-gravity jellyfish-like swimmers (*14*, *15*, *17*–*20*); in this context, we only compared jellyfish-like robots that entirely depended on active propulsion for upward swimming rather than floating based on density change (*16*). Moreover, given the electrohydraulic actuation without intermediate motion transmission mechanisms and jellyfish-like efficient locomotion, fast swimming is accompanied by relatively high energy efficiency. The robot has a low cost of transport (CoT), which is around 19.5 J/kg·m even at the fastest propulsion mode (Fig. 2D; details are given in table S3). Here, to allow a direct comparison with the previous studies on animal locomotion (*9*), we used CoT to evaluate energy efficiency. To this end, we estimated the input electrical power *P*_in_ according to the peak output voltage of the HV generator (Φ_max_ = 6.5 kV), and we did not consider the energy loss in the circuits, following a previous study (*43*) (see the “Analysis of the energy efficiency” section in Materials and Methods). The detailed robot structure could be further optimized to enhance the swimming performance by adopting a lower-drag design, e.g., by changing the six cylinder–shaped air pockets in the buoyancy unit into a single ellipsoid–shaped design. Note that we only compared the propulsion speed and CoT of the lappet paddling–based jellyfish-like swimmers. Although the pure jetting-based squid-like swimmers, which were actuated by changing the volume of the cavity, could possibly achieve a higher *v* (*34*, *44*), the continuous membrane and coupled actuation design would limit the robot’s ability to individually actuate each lappet for different underwater functions, as we will discuss in the later sections.

To investigate the robot locomotion capabilities in a more dynamic environment, we have added an external disturbance, which provided the speed of the flow of 30 cm/s in the experiment tank (fig. S6A) using a hydraulic pump (Eden multifunction pump 105). While the variance in the distance to the flow source from 15 to 35 cm led to significant differences in the maximum achieved tilting angle γ_tmax_ [*P* = 9.3 × 10^−5^, one-way analysis of variance (ANOVA) test], it did not cause significant differences in the recovery time *t*_r_, i.e., the time for *γ*_t_ recovered from γ_tmax_ to 0°, which was around 2.0 s (*P* = 0.45, one-way ANOVA test). We could observe that the stabilization unit assisted the robots in recovering back to the initial direction of upward propulsion.

Using the HASEL jellyfish robotic platform, we could generate bioinspired motions with high propulsion speed and low energy cost, both of which are among the best jellyfish-like robotic swimmers in the literature. However, the CoT for the robot presented here (more than 10 J/kg⋅m) is still not comparable to biological systems, where *A. aurita* achieves a CoT below 1 J/kg⋅m (*9*). To improve CoT, we could first upgrade the robot’s design and control strategies, such as optimizing the shape of the buoyancy unit to lower the fluidic drag and optimizing the motion sequences such that the jellyfish-like nonreciprocal motion, which has shown a critical role in enhancing energy efficiency (*9*), could be properly emulated. Moreover, the circuits could be further developed to overcome the low energy conversion efficiency in the current prototype. Last, the material used as the elastic layer to provide the restoration force and as a waterproof layer to avoid the connection between the HV electrode and ground electrode, VHB, is a typical viscoelastic material (fig. S5), where cyclic loading and unloading results in mechanical energy being dissipated as heat; replacing VHB with a more elastic material will enhance efficiency.

Another desirable property for underwater robots is to be noise free. Robots with a low sound level can safely interact with various biological species without causing harmful effects. Conventional actuation methods, such as electric motors, usually generate electrical and mechanical noise from transmissions. Similarly, for hydraulic pumps, besides the noise from the inner electrical motors, the mechanical components in the pump and the fluctuation in the fluid pressure also generate notable noise during a cyclic operation. We used two commercial actuators for underwater robots, servo motors (Parallax Inc.) and hydraulic pumps (Eden 105), and measured their sound level during operation through a decibel meter positioned just above the water surface. Regardless of the depth that these actuators were placed underwater, sound levels up to around 63 dB could be clearly differentiated from the background, which was around 53 dB (Fig. 2E). Finely engineered versions of these actuators could mediate noise levels. However, these revisions would require demanding fabrication efforts and extra components. These inevitable anthropogenic noises during the operation of underwater vehicles are a growing threat to the inhabitants of the waterbodies, ranging from minor, temporary shifts in behaviors to immediate death (*45*). In contrast, the HASEL actuators we used only produced sound at around 53 dB. The HV driving circuits were directly placed beside the water tank during sound level measurements. The sound level of the entire system, including the robots and the circuits, could barely be differentiated from the background without the need to make any revisions to their design (Fig. 2E). Therefore, this robot design may inspire future types of noise-free underwater vehicles.

### Contactless object manipulation and fluidic mixing

**Fig. 3.**
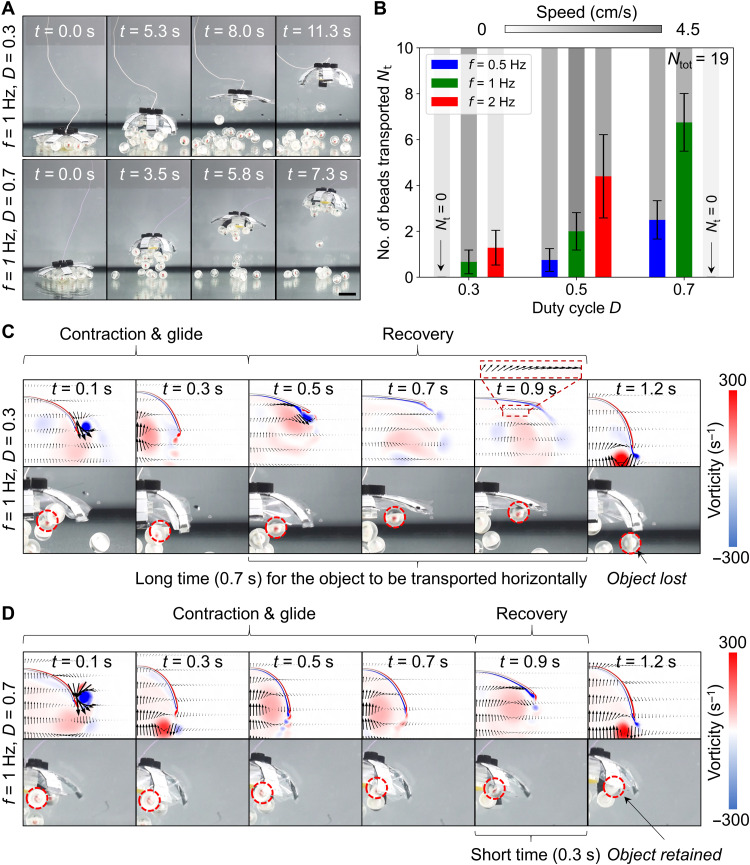
Contactless object manipulation. (**A**) Experimental snapshots at two different actuation signals, actuation frequency *f* = 1 Hz, duty cycle *D* = 0.3 and *f* = 1 Hz, *D* = 0.7. The latter actuation signal enhanced the manipulation performance, i.e., the robot transported more beads upward. (**B**) Graph that shows the effects of *f* and *D* on the number of beads transported for more than 20 cm, *N*_t_ (movie S2). The total number of beads (the densities are 1.05 to 1.10 g/cm^3^), *N*_tot_, that were distributed under the robot’s body was 19. The robot was only successful at contactless object manipulation when *f* = 1 Hz. When *f* = 1 Hz, the relation between *N*_t_ and the propulsion speed *v* was not linear. *D* = 0.7 gave the best performance. (**C** and **D**) CFD simulation shows the robot’s capability to contactlessly manipulate objects at *f* = 1 Hz and *D* = 0.3 or *D* = 0.7, respectively. When *D* = 0.3, there was a longer recovery duration of 0.7 s. Thus, there was a higher possibility that the object would be carried horizontally to the lappet’s tip through the horizontally drifted velocity field. When a new cycle started, the objects close to the tips were propelled downward, resulting in object loss. In contrast, when *D* = 0.7, there was a shorter recovery duration of 0.3 s, and thus, there was a lower possibility that the objects traveled to the lappet’s tip through the horizontally drifted flow. The enhanced upward-drifted flow during the contraction and glide phases for *D* = 0.7 also contributed to retaining the objects. The error bars represent the SDs of the means for *n* ≥ 5 experimental trials. Scale bars, 5 cm.

As a jellyfish propels forward, the surrounding fluid enters the motion path by the pressure field and drifts along with the body. Jellyfish can use these features and control fluid flow patterns to trap preys (*13*). Similarly, our robot can also manipulate surrounding objects in a contactless manner using such fluidic flows. In this study, we investigated the effects of various *f* and *D* on the robot’s ability to collect objects and retain them during upward propulsion, i.e., manipulation. To quantify this capability, we fabricated *N*_tot_ = 19 beads using a 3D printer (Clear V4, FormLabs), each with a diameter *D*_b_ of 20 mm. We finely tuned the effective density of these beads to ρ_b_ = 1.05 to 1.10 g/cm^3^, i.e., the beads are relatively neutrally buoyant. We chose the specific dimension to be compatible with the height of the stabilization unit (note S2). The beads were randomly distributed under the robot’s umbrella-shaped body, and we counted the number of beads transported 20 cm upward (the water depth in the tank was 30 cm), denoted as *N*_t_. The experiments indicated that only *f* = 1 Hz could effectively transport beads among all the investigated *D* (i.e., 0.3, 0.5, and 0.7, as is shown in Fig. 3A and the green bars in Fig. 3B). The robot could not effectively transport beads because of the low propulsion speed *v* at *f* = 0.5 Hz and *D* = 0.3, as is indicated by the missing blue bar at *D* = 0.3 in Fig. 3B. The same happened when *f* = 2 Hz and *D* = 0.7, as is indicated by the missing red bar at *D* = 0.7. The corresponding *v* for each combination of *f* and *D* is also indicated in the figure by the grayscale bars.

We further investigated the case in which *f* = 1 Hz (green bars in Fig. 3B), and the results indicated that *N*_t_ was not linearly correlated with *v*. Although *v* was similar at *D* = 0.3 (2.8 cm/s) and *D* = 0.7 (2.5 cm/s), *N*_t_ was substantially larger in the latter case, as is shown in movie S2. This variation could be explained by the different flow field patterns generated from various *D*. These patterns were quantified by a computational fluid dynamics (CFD) simulation (COMSOL 6.0 Multiphysics), where we input the body kinematics and computed the fluid-structure interactions. When *D* was set to 0.3, the robot maintained the contraction and glide phases (HV on) for 0.3 s, and the recovery phase (HV off) occupied the rest of the 0.7 s. As shown from *t* = 0.3 s to 0.9 s in Fig. 3C, the recovery phase induced a net velocity field in a horizontal direction under the lappet, pointing away from the body. Given the long recovery durations, the beads could be easily transported horizontally to the tip, as can be seen in the experimental snapshots (*t* = 0.3 to 0.9 s in the second row in Fig. 3C). When a new cycle was started, the beads that were close to the tip could be easily ejected from the robot’s body when the lappet suddenly pushed downward. In contrast, when *D* was set to 0.7, the beads had a lower chance of being transported to the tip of the leg since the recovery phase was short (0.3 s). Therefore, the objects could be retained and transported upward (Fig. 3D). Moreover, the speed of the upward-drifted flow field was higher when *D* = 0.7 during the contraction and glide phases (which is indicated by the black arrows in *t* = 0.1 to 0.7 s in Fig. 3D), and this upward flow field carried the objects better during propulsion than when *D* = 0.3 (see the black arrows in *t* = 0.1 to 0.3 s in Fig. 3C).

**Fig. 4.**
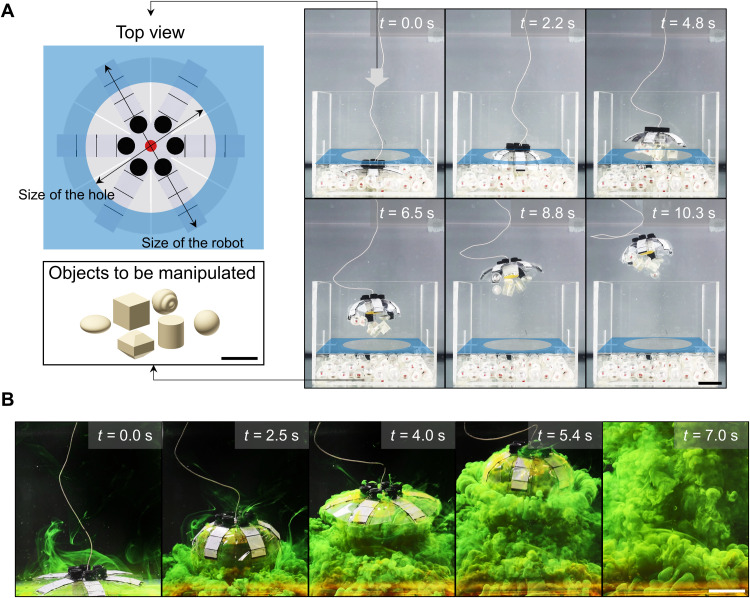
Contactless manipulation of irregular-shaped objects and dye. (**A**) Shape adaptation of the robot to transport objects of various shapes out of a confined space (*f* = 1 Hz, *D* = 0.7; movie S2). The space was composed of a box and a plate on the top with a central hole. The diameter of the hole (13 cm) was smaller than the outer diameter of the robot (16 cm). (**B**) Visualized fluidic mixing of the fluorescein dye while the robot was swimming (*f* = 1 Hz, *D* = 0.7; movie S1). Scale bars, 5 cm.

To further investigate the robot’s ability to transport objects in a contactless manner, we fabricated more samples with various shapes, including cylinders, ellipsoids, and other randomly shaped objects. We distributed them randomly under the robot and placed them at the bottom of a plastic box. A board covered the box with a hole in the center, and the hole’s diameter was 13 cm, which was smaller than the robot’s diameter of 16 cm. The experiments indicated the robot’s capability in shape adaptation, i.e., it could adaptively change its body diameter cyclically during the propulsion and pass the barrier to transport objects of various shapes (Fig. 4A and movie S2). These results suggest that this function could potentially be practical to sample fragile objects with a similar density as the water in complex and confined underwater spaces, e.g., fish eggs (around 1.02 g/cm^3^) in coral reefs (*46*). Moreover, according to the range of the drifted flow, we expect that the size of the objects to be transported should be within the robot’s dimension.

Similarly, the drifted flow induced by the jellyfish-like upward propulsion could also move the higher-density water at the bottom of vertically stratified fluid to the upper side of waterbodies. According to Darwin’s mechanism for biogenic mixing, this phenomenon eventually enables fluid mixing across all layers of the waterbodies (*47*). We explored this capability using fluorescein dye (fluorescein sodium, Fisher Chemical), which we laid under the robot’s body and placed on the bottom of the tank. The robot was then actuated with an HV signal of *f* = 1 Hz, *D* = 0.7. It was observed that the dye traveled along with the robot as it propelled. Ultimately, the dye was mixed in the tank after only 7 s (Fig. 4B and movie S1). This function could be used for biomixing, which is critical for maintaining marine circulation and transporting nutrients across all layers of waterbodies.

To gain a deeper understanding of the capability of contactless manipulation, we compared our robot with the millimeter-scale magnetic jellyfish robot by Ren *et al.* (*13*) regarding the effects of contactless object manipulation. In short, the performance of the two platforms is very comparable. First, we quantitatively compared (i) the maximum number of transported objects, (ii) the size of the transported object (relative to the body dimension), and (iii) the maximum distance of object transportation (relative to the body dimension) in both studies. The results are summarized in table S4. Moreover, the strategies to achieve better contactless manipulation is similar on both platforms. Ren *et al.* (*13*) reported that a relatively small amplitude of the body contraction during upward propulsion was suitable for long-distance object transportation. Similarly, we have discovered that a relatively shorter recovery duration, i.e., a smaller amplitude of the body deformation in each cycle, was more beneficial. With these similarities, we infer that contactless object manipulation should be practical for millimeter-scale to centimeter-scale robots operating in the transitional region from intermediate to inertial flow regime.

The above phenomenon could be explained by fluidic flow patterns generated from the fluidic-structure interaction during the robot locomotion, e.g., what is the range of the fluidic field, whether turbulent flow or laminar is more dominant, and how the flow field evolves with time. First, compared with the millimeter-scale robot, the centimeter-scale one with higher speed could contribute to a stronger and larger range of flow fields for contactless manipulation, tending to bring more and larger objects with the drifted flow. On the other hand, the centimeter-scale robot operates from the intermediate to the inertial fluidic regime (*Re* ≳ 2000), and the fluidic field could be more dominant by the turbulent flow (*48*). Such flow patterns are random and hard to predict precisely. In contrast, the millimeter soft jellyfish robot operates in the intermediate flow regime (1≲ *Re* ≲ 2000), where the fluidic field becomes laminar and is less affected by turbulence. Consequently, the field is less random and easier to predict, which might favor steady contactless object transportation for longer distances. Moreover, unlike the reciprocal motion sequences used in the centimeter HASEL jellyfish robot, the millimeter robot uses nonreciprocal motion (*13*), which could be beneficial for manipulation capability (*9*). In summary, with the variations of the flow patterns across different scales, the robots operating in the transitional regions from intermediate to inertial regime could balance the effects of the manipulated flow field ranges and the turbulent flow, which is suitable for contactless object manipulation. Certainly, for more systematic comparison and to further validate the conclusions above, robots with the same design, materials, and fabrication strategies but with different scales across millimeters to centimeters need to be analyzed in future studies.

### Diverse functions enabled by individual lappet control

**Fig. 5.**
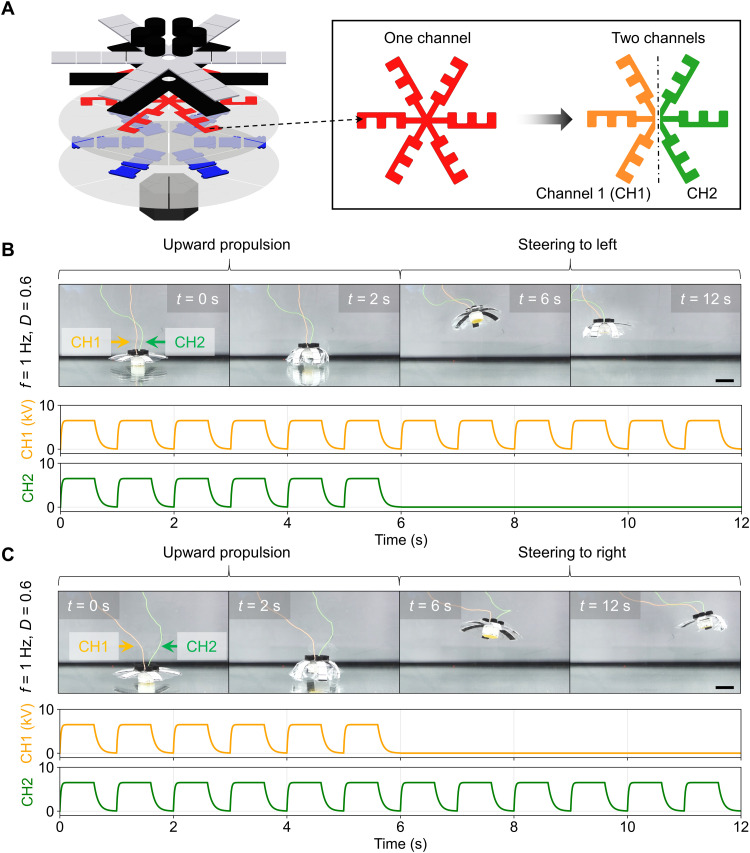
Steering enabled by separately actuated lappets. (**A**) Two independent channels of the electrode, i.e., CH1 and CH2. Each channel can operate the corresponding lappets with its own actuation signal. (**B**) Robot steering to the left and the corresponding actuation signals of the two channels (*f* = 1 Hz, *D* = 0.6). (**C**) Robot steering to the right and the corresponding actuation signals of the two channels (*f* = 1 Hz, *D* = 0.6; movie S3). Scale bars, 5 cm.

Direct electrohydraulic actuation occurs in our robot design without needing any intermediate bulky motion transmission mechanisms. Thus, it is practical to individually control each lappet so they can be used for diverse functions. The first function that we explored was steering. We divided the HV electrodes into channels 1 (CH1) and 2 (CH2), as shown in Fig. 5A, so each segment could be actuated individually. By simultaneously applying the same cyclic signals to two channels, the lappets could deform synchronized and propel upward, as seen above. On the other hand, by applying a cyclic HV to only three electrodes on one channel and stopping actuation on the other channel, the robot could be steered to the left or right (Fig. 5, B and C, and movie S3). On the basis of quantifying the steering and sinking speeds (fig. S7A) on top of the propulsion speeds, we have controlled the robot to follow an “M-shaped” path, as seen in fig. S7B.

**Fig. 6.**
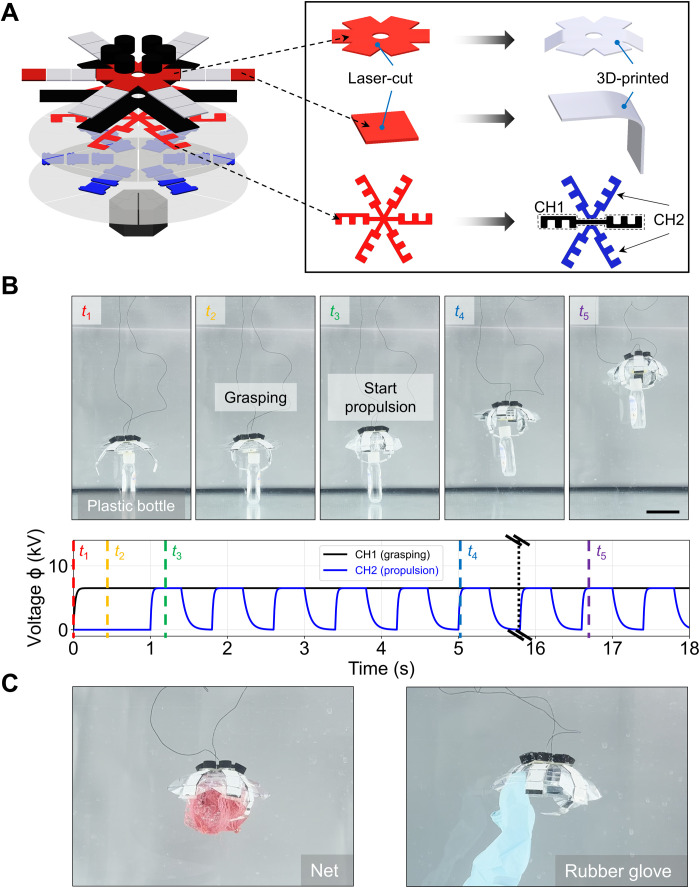
Grasping during propulsion enabled by separately actuated lappets. (**A**) Revised components and two separate electrode channels for grasping during propulsion. CH1 was actuated with a constant signal for grasping, while CH2 was actuated with a cyclic signal for propulsion. The stiffening layer was replaced with a 3D-printed one with a curved shape above CH1. This made it possible for the two gripper lappets to be bent down at the rest state for the convenience of grasping. The stiffening layers at the tip of the gripper were updated to a 3D-printed version with a curved shape. This revision ensured that the exerting forces would point to each other when grasping. (**B**) Grasping a plastic bottle (effective density, 0.90 × 10^3^ to 1.10 × 10^3^ kg/m^3^) during propulsion and the corresponding actuation signals of two channels (*f* = 1.25 Hz, *D* = 0.5 for CH2). (**C**) Two more examples (net and rubber glove with effective density of around 0.90 × 10^3^ to 1.10 × 10^3^ kg/m^3^) of the robot grasping different objects while propelling (movie S4). Scale bars, 5 cm.

The second function we achieved using individual lappet control was grasping objects during propulsion. Here, we took a 3D-printed top stiffening board (Clear V4, FormLabs) that was designed with an initial curved shape, and we altered it by enlarging the bending angles of the two corresponding diagonal lappets at the rest phase. The electrodes were also divided into two channels. CH1 included the two diagonal electrodes and was supplied with a constant HV signal, functioning as a gripper. CH2 included the rest of the four electrodes, and it was supplied with a cyclic HV signal, functioning as the lappets for propulsion (Fig. 6A). The constant HV signal was supplied to grasp the object first, and then the cyclic HV was supplied 1 s later (Fig. 6B). We demonstrated that marine trash, e.g., a plastic bottle, a net, and a rubber glove, could be transported upward (Fig. 6C and movie S4). This function could help deliver objects that are not fragile and need to be firmly gripped by the robot with little possibility of escaping, e.g., marine litter with various densities, sizes, and shapes.

To further validate the effectiveness of the grasping, we have quantified the grasping force exerted on objects of different sizes (fig. S8). The results showed that with the size increasing, the maximum normal force applied to the object increased to and saturated at around 25 mN. However, given the diversity in the objects’ shapes, surface properties, and possible dynamic flow in realistic application scenarios, the grasping provided by the closure of two lappets could be insufficient. Therefore, we have looked into potential solutions to improve the effectiveness of grasping and propulsion for object transportation. We observed that extra adhesives could help improve transportation capabilities when the robot is exposed to external disturbance (fig. S9). Thus, effective underwater adhesives could be integrated into our platform to improve future real-world utility (*49*).

Please note that a single robot could not carry objects with much higher effective densities than water due to using two lappets for grasping rather than propulsion. There are multiple feasible approaches to enhance such a capability. One possible solution would be to separate the grippers from the propellers, place them on the bottom, and use all six lappets as propellers to enhance the thrust. Second, practical approaches from other established research fields, such as buoyancy-tunable mechanisms (*11*, *35*, *36*)*,* could be incorporated into our platform for much enhanced loading-carrying capability. For example, after the robot approaches the targeted location efficiently and grasps the objects, inflation of the buoyancy-tunable unit by heating up the low–boiling point liquid inside can notably increase the buoyancy force for the effective upward transportation of real-world trash. Last but not least, two or more robots could form a team to transport objects that are too heavy for one robot, as shown in the next section.

To better understand the effects of the transported objects on locomotion efficiency (quantified by CoT), we conducted additional experiments and analyses. As shown in fig. S10A, for the three objects with a similar effective density as water, i.e., nets, glove, and plastic bottle (detailed properties are summarized in table S5), the resultant CoTs were not as low as the ones of free swimming without objects being transported. However, the values were still smaller than the ones of most jellyfish robots in literature without the ability to transport objects, as summarized in Fig. 2D and table S3, which were powered by hydraulic pumps (*15*), SMAs (*17*–*19*), and DEAs (*20*, *21*). The above results indicated the benefits of efficient object transportation using our platform. Another critical observation is that the resultant CoTs are object-dependent and orientation-dependent. A primary factor leading to such dependences is the projection area of the objects facing the propulsion direction (fig. S10B). A larger area leads to higher drag force, slows the robot, and notably increases the CoT. For example, the transportation of the horizontally placed glove with a larger projection area induced higher CoT than the vertically transported glove with a smaller area.

One of the unique benefits of using individually controlled HASEL actuators for diverse functions is that increasing the degrees of freedom in terms of actuation does not increase the overall size of the control electronics. For example, two hydraulic actuators are needed to realize the left and right steering as a conventional approach (*15*). More actuators or complex motion transmission mechanisms would be required if we need more steering directions, notably increasing the robot’s size. In contrast, on the basis of recent progress, 10-channel individually controlled outputs could be generated by a cell phone–sized HV power supply, enabling individual controllability of all six lappets on the robot for diverse functions while keeping the lightweight system (*50*). Such a capability could improve the robot’s agility for more than moving up for manipulation/transportation of the objects from the bottom to the surface in the waterbodies toward potential applications such as recycling marine trash.

### Cooperation of two to three robots and a wireless robot prototype

**Fig. 7.**
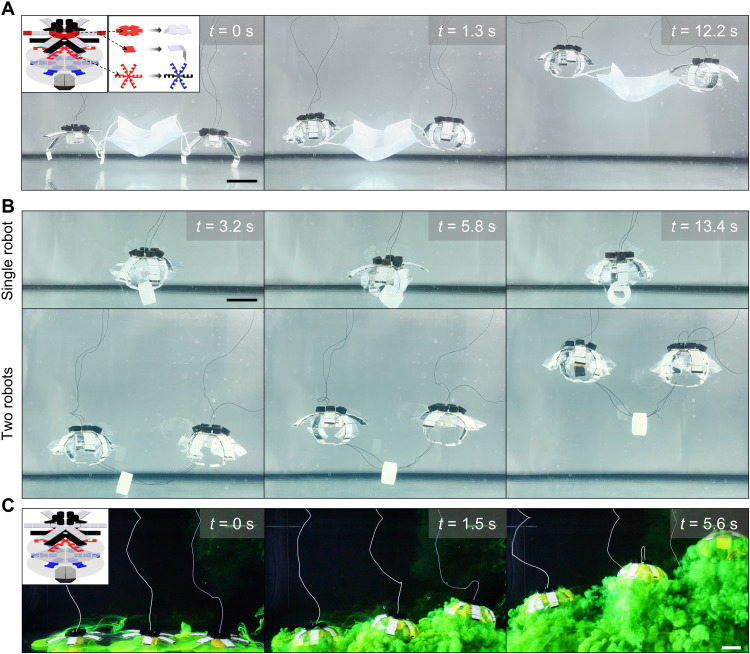
Tasks performed by individually controlled robots. (**A**) Grasping of a mask and propelling by two robots (*f* = 1.25 Hz, *D* = 0.5; movie S6). (**B**) Example of transporting the scotch tape with a substantially larger density than water (2.08 × 10^3^ kg/m^3^), where only one robot failed to transport (*f* = 1.25 Hz, *D* = 0.5; movie S6). (**C**) Fluidic mixing by three robots. The robots were sequentially launched (*f* = 1 Hz, *D* = 0.7) with a delay of 1 s (movie S7). Scale bars, 5 cm.

Just like jellyfish blooms mix the ocean and contribute a massive amount of energy to aquatic ecosystems (*47*), several HASEL jellyfish robots could enhance object manipulation tasks. First, they could transport objects in a contactless manner, rendering them efficient at collecting fragile samples. As is shown in movie S5, *N*_t_ = 12 objects of various shapes were transported by three individually controlled robots. These robots were launched in sequence with a time delay of 1 s. Moreover, two or more robots could cooperate to deliver objects that are hard for an individual robot to grasp and transport. For example, two robots could simultaneously grasp the two mask leashes, propel themselves, and transport the scotch tape together, whereas only one robot failed to finish this task (Fig. 7, A and B, and movie S6). We have quantified the CoTs for the transportation of the mask and the scotch tape by two robots. The values are also around 100 J/kg·m and are still smaller than most jellyfish robots without the ability to transport objects reported in the literature. Actually, the only jellyfish robot showing lower CoT than our platform when transporting objects was designed at a meter scale, powered by noisy linear motors, used rigid motion transmission mechanisms, and did not show the capabilities of steering and object manipulation (either contact-based or contactless) (*14*). The above experimental results have shown the feasibility of using the HASEL jellyfish robot platform for transporting more than small and light objects with relatively low energy costs. The possible integration of a buoyancy-tunable mechanism could enhance load-carrying capabilities, and it will be critical to investigate whether it could also improve the efficiency when transporting objects with large projection areas and effective densities. Last, we demonstrated that three robots could also be used to mix fluid, potentially enhancing the circulation of nutrients and other dissolvable materials even at a larger scale (Fig. 7C and movie S7).

**Fig. 8.**
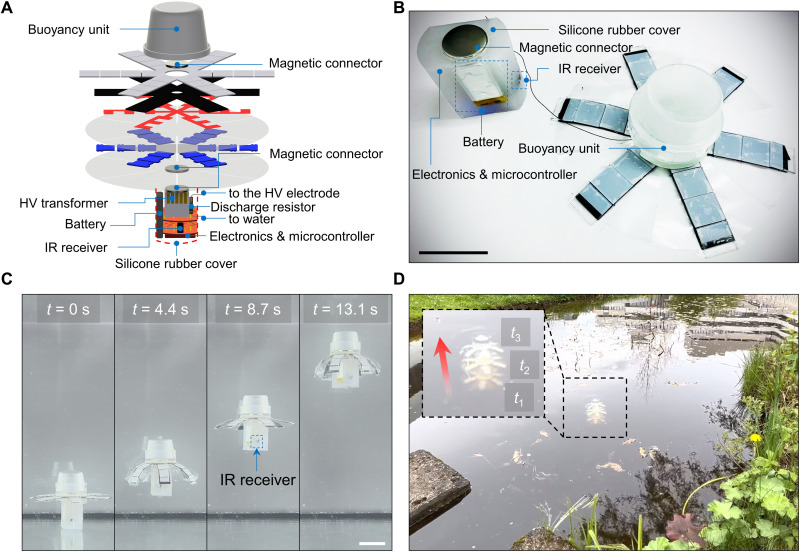
A prototype of a wireless HASEL jellyfish robot. (**A**) Design details with a computer-aided design (CAD) image. (**B**) A snapshot of the wireless robot. The prototype was equipped with all electronics onboard, including circuits for HV generation, an infrared (IR) receiver, and batteries. The robot was remotely launched and stopped by a remote infrared controller. (**C**) Propulsion test in a water tank (*f* = 1.25 Hz, *D* = 0.5; movie S8). The robot could operate for 1 hour using a commercial battery (lithium-ion polymer battery; capacity, 350 mAh; nominal voltage, 3.7 V). (**D**) Propulsion test in a pond outdoors (*f* = 1.25 Hz, *D* = 0.5; movie S8). Scale bars, 5 cm.

To apply HASEL jellyfish robots to real-world scenarios, we prototyped a fully wireless version for future autonomous environmental applications. All electronics, including an infrared receiver for wireless remote control and batteries (lithium-ion polymer battery; capacity, 350 mAh; nominal voltage, 3.7 V; weight, 12 g), were integrated and waterproofed by silicone (Ecoflex -00-30, Smooth-On Inc.) (Fig. 8, A and B; see the “Hardware and software to generate and control the HV” section in Materials and Methods). An infrared controller remotely launched the robot. We tested the wireless robot in the water tank and a pond outdoors (*f* = 1.25 Hz, *D* = 0.5) (Fig. 8, C and D, and movie S8), and the current prototype could operate continuously for more than 1 hour. The characterization showed that the wireless prototype achieved an average propulsion speed of 1.97 cm/s and CoT of 15.88 J/kg·m at *f* = 1.25 Hz, *D* = 0.5. A detailed comparison with the wired version is given in table S6.

As seen above, on the basis of the analyses, the CoT for the wireless version was smaller than the wired version’s. This phenomenon aligned with a previous study where the wireless DEA-powered jellyfish-like robot achieved lower CoT than the wired version (*20*). The results of our study could be explained from two aspects. First, from the calculation aspect, given the similar input power, the mass of the wireless version is more than three times larger than that of the wired version, but the speed is half that of the wired version. Consequently, the CoT of the wireless version is smaller than the wired version (table S6). Second, the top of the wired version is occupied by six symmetrically distributed buoyancy units that protrude from a flat plate, which notably increases the fluidic drag. On the other hand, the buoyancy unit designed for the wireless version has a single part and a smooth shape like an ellipsoid, which could be more beneficial to reduce the drag and help enhance the locomotion-relevant energy efficiency. Therefore, structural optimization should improve the robot’s efficiency further.

The current wireless prototype could also manipulate objects in a contactless manner (fig. S11). However, it currently has one channel with an HV output of Φ_max_ = 6.2 kV, and the overall electronics part, around 5 cm in height, could potentially hinder the interaction with the objects for effective manipulations. Each lappet could be individually controlled using a multiple-channel HV power supply with miniaturized electronic components to minimize the negative effect on object manipulation and other functions (*50*).

## DISCUSSION

To tackle the limitations of the state-of-the-art underwater robots in achieving noise-free and gentle interactions with underwater environments along with other practical functions, we have developed a centimeter-scale deformable and adaptive jellyfish-like robotic platform. The prototype provides noise-free operations and gentle manipulation of objects without physical contact to interact with the underwater ecosystem safely. Despite being driven by HV signals, only low currents at the microampere level are used for operation, well below the level of safety standards suggested by UL and the IEC (*42*). Even in the worst-case scenario of direct exposure of HV signals to surrounding water or the human body, the acoustic and electrical properties of the robots remain harmless (note S3) (*42*, *51*–*53*). As a result, the HV actuation principle poses no danger to marine wildlife. While our platform also harnesses the widely adopted jellyfish-inspired architecture, our platform has accomplished superior performance in both propulsion and multifunctionality by exploiting a hybrid structure comprising both rigid and soft components and by enabling individual lappet control. Our design offers a platform for further development towards the next generation of safe underwater vehicles. Furthermore, like jellyfish blooms contribute a massive amount of energy to marine ecosystems, it can be expected that the collective operations of a swarm of HASEL jellyfish robots, in which each robot would be individually controlled, would help underwater environmental remediation and facilitate the circulation of nutrients at a large scale (*47*, *54*).

However, several essential aspects must be addressed before translating this system into fully autonomous vehicles for field operations. First, given the platform’s usage in natural habitats, the materials for the platform need to be improved to boost robustness, reliability, and longevity in underwater environments (*55*, *56*). Two solutions could be adopted to overcome the polyethylene terephthalate (PET) film’s damage resistance to sharp objects and to mitigate the potential negative effects of the consequent leakage of the liquid dielectric. First, replacing the polymer shell with resilient materials will improve the robustness and life cycle of the robot. Recently, silicones resilient to damage have been used to fabricate underwater robots leveraging HV actuation (*37*, *43*), and self-healing polymers are being introduced (*57*). The ones suitable for fabricating HASEL actuators, such as Ecoflex 00-30 (*28*), could be adopted. Second, biodegradable materials may be explored to mitigate the potential damage to the environment. There are efforts for biodegradable materials used for soft robots (*58*), including polymers, electronics, and batteries, that can be integrated into our platform. For example, the biodegradable liquid dielectric (Envirotemp FR3, Cargill) readily applies to HASEL actuators (*28*).

Concerning the longevity of the platform, the occurrence of dielectric breakdown and the lifetime of HASEL actuators underwater depend on many factors, including the maximum electrical field, the composition of the surrounding water, the defect density of commercial PET film, which here is intended for food packing and not fully optimized for the use in electrostatic devices, and the rate of usage of the robot. Generally, with the actuation signal of *f* = 1.25 Hz, *D* = 0.5, Φ_max_ = 6.0 to 6.5 kV for around 10 cycles of swimming (from tank bottom to the water surface) in one experiment trial, which was repeated for at most 20 times a day, the robot could survive over 2 weeks without breakdown. However, if we further increased the rate of usage per day, the robot could break down within a day, and thus, the overall lifetime dropped. Experimentally, we observed that the dielectric breakdown usually happens through the heat seal used to create the desired shapes of pouches. This phenomenon may be related to the repeated partial discharges through the heat seal leading to electrical degradation and subsequent breakdown (*30*). There are several solutions to overcome such a limitation and improve the lifetime. First, the drawback could be mitigated using self-clearing electrodes that insulate the breakdown region (*59*). Second, dielectric coatings could be implemented at the heat seals to enhance their strength (*60*). Third, using adhesives instead of heat seals to form the polymer shells could prevent premature electrical failure of the heat seals. Fourth, HASEL actuators based on elastomers could be adopted, which have shown a high lifetime in previous investigations (*30*). Last, we anticipate that developing new materials with customized properties will allow for a substantially enhanced lifetime, such as thin films that combine self-healing abilities, high dielectric constant, high dielectric breakdown strength, mechanical strength, and flexibility.

Further, our robotic platform will benefit from more capable electronics, such as a more powerful central processing unit, miniaturized circuits to control each lappet individually, various added sensors, and a customized battery. For example, more efficient circuits for HV generation should be developed. Although our current circuits can realize various required tasks, they are not yet designed to be energy efficient; our calculations indicated that the energy conversion efficiency from the low voltage side (5 V) to the HV side (6.5 kV) is below 20%. Innovation in this direction will substantially improve operational time on a single battery charge. Moreover, the platform should withstand high pressure for potential environmental applications in benthic territories. A promising solution is to decentralize the electronics and integrate them into a silicone matrix (*37*). On top of the systematic improvements at the component level, the impacts of the optimized platform on the underwater ecosystem must be evaluated. For example, permission to conduct quantitative investigations on the interactions with different underwater species needs to be requested from animal facilities following applicable local laws.

With the above advancements, improved control and planning algorithms could be studied to enable other critical scientific investigations and applications. For example, the effectiveness of a swarm could be studied. In nature, aggregated animals might interact with each other by manipulating flow fields, e.g., boundary layers of the drifted volume or vortices shed from another swimmer in the school (*47*), to enhance collective energy efficiency and biogenic mixing. It would be possible to study such interactions using our platform, given the individual controllability of every single robot; exciting scientific questions could be investigated related to mutual interactions of individual robots and their impact on the swarm’s locomotion performance and overall functionality. To implement such controlled team operations, communications among HASEL jellyfish robots are essential. To this end, the available techniques, including acoustic waves, optical wireless communications, electromagnetic waves, magneto-inductive communications, and electric currents, could be adopted. Specifically, optical communication could best serve the function when the robots operate in proximity to each other (*61*, *62*). Such studies will benefit the advancement of both the exploration of special underwater environments and the field of autonomous mobile robots that operate in complex and unstructured environments.

## MATERIALS AND METHODS

### Fabrication of the HASEL jellyfish robot

The detailed fabrication process is shown in fig. S1. The overall robot consists of an actuation unit, a power and control electronics unit, and a buoyancy-stabilization unit. We fabricated the actuation unit layer by layer in a 2D manner. First, we sealed two polymer films (Mylar 850, 15 μm, Petroplast GmbH) using heat to form the designed shell shapes for the HASEL joints. Next, we printed a layer of carbon ink (Cl-2051, Nagase ChemteX America Corp.) on top of the film. This layer of ink worked as the HV electrode during actuation. Next, we covered the electrode with a waterproof layer of double-sided adhesive tape (VHB 5925, 3M). This layer also functioned as an elastic material by providing restoring force during deformation. We then filled the shells with liquid dielectric (silicone oil, 5 cSt, Sigma-Aldrich Ltd.). The design parameters can be found in table S1. On top of the waterproof layer, we attached copper wires, which were covered with silicone, to the electrode using conductive copper tape and waterproofed the connection part with PDMS (Sylgard 184, Dow Inc.). Last, we attached a stiffening layer made of 1-mm-thick acrylic boards. In the power and control electronics unit, for the wired version, we connected the copper wires to the off-board electronics and power supply. In the wireless version, we connected the copper wires to the onboard electronics, including the battery, which were attached to the bottom of the robot. Since there were minor differences in the volume of oil that was injected into each lappet, and the HV cable caused random forces applied on the robots, the robots tended to flip and reorient during upward propulsion. To stabilize the robot, we arranged a stabilizing-buoyancy unit. For the stabilization unit, we used a mass block that weighed around 26.5 g to make the center of mass as low as possible in the wired version. The onboard electronics attached to the bottom were used as an additional mass for the wireless version. Then, we attached a buoyancy unit to the robot’s top to tune the robot’s density. With this stabilizing-buoyancy unit, the resultant effective densities for the wired and wireless robots were around 1.23 g/cm^3^.

### Hardware and software to generate and control the HV

We generated the desired HV using a two-stage circuit. The first stage was a flyback circuit, which boosted the power supply’s (or battery’s) voltage from 5 V (or 3.7 V) to an amplitude of around 1 kV. Here, a 20-kHz, 30% duty cycle pulse signal was used, and it was regulated by the microcontroller. In the wired version, we used Arduino Uno as the microcontroller, and it communicated with the PC through ROS for control purposes. In the wireless version, we used DFR0282 Beetle (DFROBOT) as the microcontroller, and the program was preloaded before the electronics were waterproofed in silicone rubber. The second stage was the multiplier circuit, which boosted the maximum output voltage to around Φ_max_ *=* 6.5 kV for the wired version and 5 kV for the wireless version. Except for the demonstration of a wireless prototype’s swimming, in all other quantified investigations, we unified the amplitude to Φ_max_ *=* 6.5 kV for actuation, and we operated the robot with an output frequency *f* ranging from 0.25 to 2 Hz. The details of the electronics design are shown in fig. S3A, and the bills of materials for the electronics are shown in table S2. Since the HASEL actuators could be considered serial connections of a resistor (estimated to be *R*_r_ = 333.3 ohms) and a capacitor (estimated to be *C*_r_ = 2.3 × 10^−9^ F at the fully actuated state) (table S1) (*41*), the robot’s integration will influence the time response of the circuits’ HV output Φ. As shown in fig. S3B, we experimentally decided the robot’s charge time *t*_c_ and discharge time *t*_d_, which are 0.1 and 0.3 s, respectively. Please note that this is the circuits’ property and does not vary with different actuation parameters *f* and *D* (fig. S3C). The software for communicating with the PC was built on the basis of the ROS Melodic Morenia. To start the robot, we used a customized launch file within the ROS environment for the wired version, and we integrated an infrared receiver into the electronics to realize the remote control for the wireless version.

### Analysis of the energy efficiency

The energy efficiency of jellyfish-like swimming can be evaluated by the CoT or mass-specific energy input per distance traveled (*63*), and in this study, we used CoT (*4*, *20*) given by

$$\mathrm{CoT}=\frac{E_{\mathrm{in}}}{m_{r}d}=\frac{P_{\mathrm{in}}}{m_{r}v}$$ (1)

where *E*_in_ and *P*_in_ are the input electrical energy and power consumption from the HV generator, respectively; *m*_r_ is the mass of the robot; and *d* and *v* are the displacements and average speed of the robot along the global *z* axis, respectively. We considered the conversion from electrical energy to kinetic energy and neglected the losses in the circuits for voltage amplification, following previous studies (*20*, *43*), where *P*_in_ could be expressed as

$$P_{\mathrm{in}}=C_{\mathrm{act}}\phi^{2}Df$$ (2)

where *C*_act_ is the capacitance of all HASEL actuators at the full actuated states, Φ is the amplitude of the applied voltage, *f* is the actuation frequency, and *D* is the duty cycle. *C*_act_ can be expressed by (*41*)

$$C_{\mathrm{act}}=\epsilon_{0}\epsilon_{r}\frac{wh}{2t_{m}}$$ (3)

The values of relevant parameters can be referred to in table S1. We took the data after the robot was launched for three cycles to analyze energy efficiency.

### Dynamics model of a swimming HASEL jellyfish robot

The modeling was subdivided into two steps: modeling the body kinematics from the input HV, i.e., the joint rotation angle θ_1_-θ_3_ and the angular speed ${\dot{\theta }}_{1}$-${\dot{\theta }}_{3}$, and modeling the upward propulsion performance from the body kinematics. For the first step, we used the Lagrangian equations of the second kind to derive the equations of motion for the joints by

$$\frac{d}{dt}\left(\frac{\partial A}{\partial \dot{\alpha }}\right)-\frac{\partial A}{\partial \alpha }=q_{v}+q_{d}$$ (4)

where *A* = *T* – *P* is the Lagrangian of the system (*T* is the kinetic energy and *P* is the potential energy), *q*_v_ is the generalized force due to viscous dissipation, and *q*_d_ is the generalized force due to the fluidic drag that was applied to the rotational link. The modeling details can be found in note S1.

For the second part, we used the body kinematics that had been computed from the above equation and followed the hydrodynamic model for jellyfish swimming (*63*, *64*)

$$T+D+R=m_{r}\frac{d^{2}z}{dt^{2}}$$ (5)

where *T* is the thrust force, *D* is the drag force, *R* is the acceleration reaction force, *m*_r_ is the robot’s mass, and *z* is the robot’s upward displacement against gravity. *T* can be expressed as (*64*)

$$T=\left(\frac{\rho_{w}}{A_{s}}\right){\left(\frac{dV_{s}}{dt}\right)}^{2}$$ (6)

where ρ_w_ is the density of water, *A*_s_ is the instantaneous projected area of the sub-umbrella opening, and *V*_s_ is the volume of the sub-umbrella cavity. *T* is applied in the opposite direction from that of the ejected fluid. *D* can be expressed as

$$D=\frac{1}{2}c_{d}\rho_{w}A_{s}{\dot{z}}^{2}$$ (7)

where *c*_d_ is the drag coefficient of the robot. *D* is applied in the opposite direction of the robot’s movement. *R* can be modeled by

$$R=\alpha \rho_{w}V_{s}\frac{d^{2}z}{dt^{2}}$$ (8)

where α = (2*h*_t_/*d*_t_)^1.4^ is the added mass coefficient, *h*_t_ is the bell’s height, and *d*_t_ is the bell’s diameter. The dynamic models were solved by the ODE45 solver in MATLAB R2020b (MathWorks Inc.) for 10 cycles, and the average propulsion speeds *v* were computed on the basis of the later 5 cycles.

### Statistical analysis

All of the quantitative values from the experiments were presented as the mean ± SD. We used a *t* test for statistical analysis. We set the statistical significance at 95% confidence level (*P* < 0.05).

## Supplementary Materials

### This PDF file includes:

Notes S1 to S3 Figs. S1 to S12 Tables S1 to S6 Legends for movies S1 to S8
