# Supplementary material for: A versatile jellyfish-like robotic platform for effective underwater propulsion and manipulation
Source: Sci Adv. 2023 Apr 12;9(15):eadg0292. doi: 10.1126/sciadv.adg0292 (PMC10096580; doi:10.1126/sciadv.adg0292)
Supplement: Supplementary file 1 — Notes S1 to S3 Figs. S1 to S12 Tables S1 to S6 Legends for movies S1 to S8 [file sciadv.adg0292_sm.pdf]

Science Advances
AAAS

Supplementary Materials for

A versatile jellyfish-like robotic platform for effective underwater propulsion and manipulation

Tianlu Wang *et al.*

Corresponding author: Wenqi Hu, wenqi@is.mpg.de; Christoph Keplinger, ck@is.mpg.de;
Metin Sitti, sitti@is.mpg.de

Sci. Adv. **9**, eadg0292 (2023)
DOI: 10.1126/sciadv.adg0292

The PDF file includes:

Notes S1 to S3
Figs. S1 to S12
Tables S1 to S6
Legends for movies S1 to S8

Other Supplementary Material for this manuscript includes the following:

Movies S1 to S8

Note S1. Model of swimming of HASEL jellyfish robot

The full dynamic model could be subdivided into two steps: body kinematics (joint angles and velocities), resulting from the interaction between HASEL actuator-powered links and the surrounding fluid; and upward propulsion, resulting from the periodic body kinematics (**Fig. S4A**).

1.1 Body kinematics

Each joint can periodically rotate due to the electrostatic force from the applied voltage, the hydraulic pressure in the shell, the fluidic drag from the surrounding water, and the gravity of the link. First, we modeled the kinematics of a single joint. We used the geometric variable α (**Fig. S4B**) as the generalized coordinate to describe the system's configuration. Due to the highly nonlinear relations between the zipping length z and α, and because of the nonlinear relations between the joint rotation angle and the previous link θ and α, it is not feasible to explicitly express the relations as $z = f(\alpha)$ and $\theta = g(\alpha)$. We simplified the system to understand the dynamics behind fluid-structure interactions. We assumed that the actuator could linearly contract to lift the second link and bend the joint (**Fig. S4B**).

In the simplified model, we assumed that the polymer films in the liquid-filled region had cylindric sections in which half of the intersection angle was α (**Fig. S4B**). The liquid dielectric, e.g., the silicone oil with a viscosity of 5 cSt, was treated as being incompressible. Given the length of the shell L, the width of the shell w, and the volume of the liquid dielectric V, the initial angle α_0 could be computed from

$$L = \sqrt{\frac{2V\alpha_0^2/w}{\alpha_0 - \sin(\alpha_0)\cos(\alpha_0)}}. \tag{1}$$

The initial length of the actuator could be computed by

$$l_0 = \frac{L\sin(\alpha_0)}{\alpha_0}. \tag{2}$$

The zipping length z under actuation could then be expressed by

$$z = L - \sqrt{\frac{2V\alpha^2/w}{\alpha - \sin(\alpha)\cos(\alpha)}}, \tag{3}$$

and the lifting distance of the actuator x could be expressed as

$$x = l_0 - z - (L - z)\frac{\sin(\alpha)}{\alpha}. \tag{4}$$

In the current model, the actuator was assumed to be attached to the point that is $0.5r$ away from the rotational joint, i.e., the end of the shell, where r is the length of the electrode-free shell region. The joint rotation angle of link 2 with respective to link 1, θ, could be expressed as

$$\theta = \frac{2}{r}x. \tag{5}$$

The system's equation of motion could be calculated using the Lagrangian equations of the second kind:

$$\frac{d}{dt}\left(\frac{\partial A}{\partial \dot{\alpha}}\right) - \frac{\partial A}{\partial \alpha} = q_{\mathrm{v}} + q_{\mathrm{d}}, \tag{6}$$

where $A = T - P$ is the Lagrangian of the system (T is the kinetic energy and P is the potential energy), q_{v} is the generalized force due to viscous dissipation, and q_{d} is the generalized force due to the fluidic drag that was applied to the rotational link.

For the kinetic energy T, we ignored the mass of the actuator and only considered the kinetic energy of the link when it was lifted for x,

$$T = \frac{1}{2}m\dot{x}^2, \tag{7}$$

where m is the mass of the rotational link.

For the potential energy P, we neglected the electric energy stored in the electric field inside the fluid-filled region of the shell. The potential energy has four components: the potential energy from the weight; the electric energy that was stored in the zipped region of the actuator (which was treated as a deformable capacitor); the change in the voltage source's energy when the charges flowed into the actuator; and the elastic energy that was stored in the joint (i.e., the VHB tape). Thus,

$$P = mgx + \frac{1}{2}Q^2\frac{2t_{\mathrm{m}}}{\varepsilon_0\varepsilon_{\mathrm{r}}wz} - \Phi Q + \frac{1}{2}k_{\mathrm{j}}\theta^2, \tag{8}$$

where g is the gravitational acceleration, t_{m} is the thickness of the film, ε_0 is the vacuum permittivity, ε_{r} is the relative permittivity of the Mylar film, Φ is the applied voltage, Q is the charge that flowed onto the electrode, and k_{j} is the joint's rotational spring constant. Based on the relation between Φ and Q in the zipped region

$$Q = \varepsilon_0\varepsilon_{\mathrm{r}}\frac{wz}{2t_{\mathrm{m}}}\Phi, \tag{9}$$

we could simplify equation (5) to

$$P = mgx - \frac{\varepsilon_0 \varepsilon_r wz}{4t_m} \Phi^2 + \frac{1}{2} k_j \theta^2. \tag{10}$$

To model the generalized force due to viscous dissipation q_v, we followed a previous study (*41*) and approximated the flow as the Poiseuille flow between two parallel plates:

$$q_v = -24\eta\mu w \frac{\sin(\alpha)^2}{\alpha - 0.5\sin(2\alpha)} \left(\frac{\partial z}{\partial \alpha}\right)^2 \dot{\alpha}. \tag{11}$$

Here, η is the fitting factor that accounts for simplifying the flow configuration. It was chosen, based on a previous study, to be 2.85 (*41*).

To model the generalized force due to the fluidic drag q_d, we use the following expression with respect to the generalized coordinate α:

$$q_d = -\mathrm{sgn}(\dot{\theta}) \frac{1}{2} \rho_w c_d S \left(\frac{1}{2} L_2 \dot{\theta}\right)^2 \frac{L_2}{2} \left(\frac{\partial \theta}{\partial \alpha}\right), \tag{12}$$

where c_d is the drag coefficient, S is the area of the cross-section that faced the flow, L_2 is the length of link 2. We assumed that the drag force was applied at the center of, and perpendicular to, link 2.

Combining the above equations, we can get the equation of motion for the system:

$$\ddot{\alpha} + \frac{\partial^2 x}{\partial \alpha^2}\left(\frac{\partial x}{\partial \alpha}\right)^{-1} \dot{\alpha}^2 + g\left(\frac{\partial x}{\partial \alpha}\right)^{-1} - \frac{\epsilon_0 \epsilon_r \Phi^2}{4mt_m} \frac{\cos(\alpha)}{1-\cos(\alpha)} \left(\frac{\partial x}{\partial \alpha}\right)^{-1} + \frac{4k_j x}{mr^2}\left(\frac{\partial x}{\partial \alpha}\right)^{-1} = m^{-1}\left(\frac{\partial x}{\partial \alpha}\right)^{-2} (q_v + q_d). \tag{13}$$

For extensive details on the expressions for $\frac{\partial x}{\partial \alpha}$ and $\frac{\partial^2 x}{\partial \alpha^2}$, please refer to (*41*). We used the ODE45 solver in MATLAB R2020b (MathWorks, Inc.) to solve the equations above, and it gave us $\alpha(t)$. Based on equations (4) to (7), we acquired θ. Repeating the method for θ_1 to θ_3 (**Fig. S4C**), we modeled the variations of θ_1 to θ_3 over time, and the results were comparable to the measurements from the experiments (**Fig. S4D**). The models of the body kinematics captured the different responses of the three links well. Link 3 was the fastest to deform due to the smaller fluidic drag, followed by link 2 and link 1.

1.2 Upward propulsion

The time-asymmetric cyclic movement of θ_1 to θ_3 for all six lappets around the robot induced propulsion upward. This could be modeled by

$$T + D + R = m_{\mathrm{r}} \frac{d^2 z}{dt^2}, \tag{14}$$

where T is the thrust force, D is the drag force, R is the acceleration reaction force, m is the robot's mass, and z is the robot's displacement along the direction against gravity. T could be expressed according to the jet-based locomotion modes of jellyfish (*64*):

$$T = \left(\frac{\rho_{\mathrm{w}}}{A_{\mathrm{s}}}\right)\left(\frac{dV_{\mathrm{s}}}{dt}\right)^2, \tag{15}$$

where ρ_{w} is the density of water, A_{s} is the instantaneous projected area of the sub-umbrellar opening, V_{s} is the volume of the sub-umbrellar cavity. T was applied in the opposite direction from the ejected fluid. D could be expressed by

$$D = \frac{1}{2} c_{\mathrm{d}} \rho_{\mathrm{w}} A_{\mathrm{s}} \dot{z}^2, \tag{16}$$

where c_{d} is the drag coefficient of the robot. D was applied in the opposite direction from the robot's movement. R could be modeled by

$$R = \alpha \rho_{\mathrm{w}} V_{\mathrm{s}} \frac{d^2 z}{dt^2}, \tag{17}$$

where $\alpha = (2h_{\mathrm{t}}/d_{\mathrm{t}})^{1.4}$ is the added mass coefficient, h_{t} is the bell's height, and d_{t} is the bell's diameter.$\theta_1 - \theta_3$ were used to compute h_{t}, d_{t}, A_{s}, and V_{s} by

$$h_{\mathrm{t}} = L_1|\sin(\beta_1)| + L_2|\sin(\beta_2)| + L_3|\sin(\beta_3)|, \tag{18}$$

$$d_{\mathrm{t}} = L_4 + 2L_1|\cos(\beta_1)| + 2L_2\,|\cos(\beta_2)| + 2L_3|\cos(\beta_3)|, \tag{19}$$

$$A_{\mathrm{s}} = \pi\left(\frac{1}{2} d_{\mathrm{t}}\right)^2, \tag{20}$$

$$V_{\mathrm{s}} = \frac{1}{6}(\pi d_t^2 h_t). \tag{21}$$

We merged the above variables with equation (5) and solved it using the ODE45 solver in MATLAB R2020b (MathWorks, Inc.). This gave us the swimming performance of the HASEL jellyfish.

Note S2. Dimensions of the objects for contactless manipulation

In the current design, the lappets are h_l = 2 cm away from the tank's bottom at rest state before being launched due to the stabilization unit at the bottom (**Fig. S2A**). According to computational fluid dynamics, when the robot is launched, the drifted flow vector closer to the lappets is stronger in amplitude toward the upward direction than the ones far from the lappets (closer to the bottom). Thus, flow closer to the lappet makes it easier for the robot to carry the objects while it propels upward. In contrast, the flow closer to the bottom tends to point more to the body horizontally, making it harder to carry the objects upward, as shown in (a) and (b) in **Fig. S2B**. This quantified simulation also aligns with our experimental observations that the current robot configuration (with h_l = 2 cm) is ineffective at transporting objects that have an overall dimension smaller than 5 mm, i.e., the small objects that are closer to the bottom of the tank and relatively far from the lappets. Therefore, to fully utilize the flow field under the current robot and investigate the performance of contactless manipulation, we used objects with an overall dimension of around 2 cm, i.e., similar to h_l.

By decreasing the height of the extra mass on the bottom, e.g., h_l = 5 mm, it is feasible to transport objects with smaller dimensions, i.e., 1 mm – 5 mm, with an effective density of around 1.02 – 1.05 g/cm^3. This shorter height enables a closer distance between the lappets and the small objects, making it possible to carry them with the stronger upward drifted flow field (**Fig. S2C**). The robot tended to reorient during upward propulsion because the center of mass was closer to the center of buoyancy.

Note S3. Analysis of safety

3.1 Electrical hazard

3.1.1 For fish

Many factors, including the conductivity of the surrounding water, the type of electrical signal, fish species, and the fish's posture, influence the effects of electricity on fish. According to a previous report, a voltage gradient smaller than 0.1 V/cm is safe for fish (5*1*). In our current system, we simulated the breakdown by shortening the HV electrode and the ground electrode, which were 5 cm away from each other, through the water. Then we measured the voltage gradient in the water at the points where the distance from the electrodes, L, ranged from 0.5 cm to 10 cm (**Figs. S12A** and **S12B**). This simulates the exposure of the HV electrode to the water due to mechanical rupture or dielectric breakdown. The resulting voltage gradient was 9.6 mV/cm at L = 0.5 cm, which is much below the suggested safe limit of 100 mV/cm. This result implies that most fishes should be safe in the worst-case scenario of HV exposure to the water.

3.1.2 For humans

We evaluated the potential hazard for humans in the worst-case scenario where all the currents flow from the high voltage electrode through the human body to the ground electrode, and no current is distributed to the surrounding water. We simulated the situation by connecting a resistor directly between two electrodes of the wired version in the air and measured the current. We have tested different resistors with resistance ranging from 180 ohms to 5k ohms. The worst case of 180 ohms is chosen to be well below the reported resistance value of wet head-to-foot for safety margin (5*2*). The measured current through the resistor was 3.9 mA (**Fig. 12C**) which is well below the safety guideline provided by Underwriters Laboratories (UL) and International Electrotechnical Commission (IEC) (*42*). The test of wireless electronics has shown similar results.

3.2 Acoustic hazard

To investigate the sound pressure level when the robot breaks down, we have utilized the decibel meter to measure it when actuating the broken robot at different depths. As shown in **Fig. S12D**, the perceived sound level in the air could not be distinguished from the cases when the robot is normally actuated, which was around 53 dB. These measured sound pressure values in the air

correspond to around 115 dB of sound in water, which is lower than the sound of 180 dB known to cause injuries to fish (*53*).

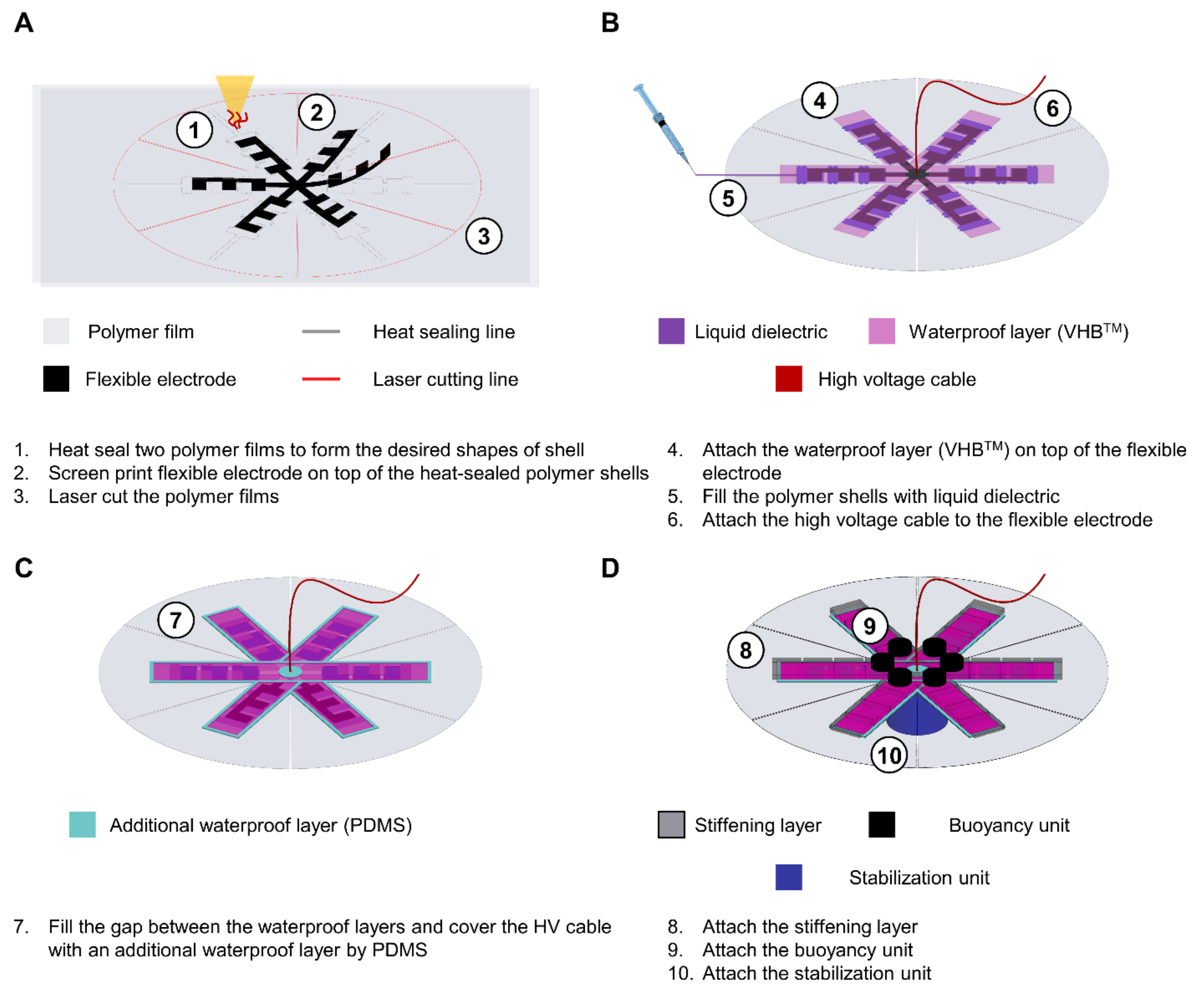

Figure S1. Fabrication details of the HASEL jellyfish robots.

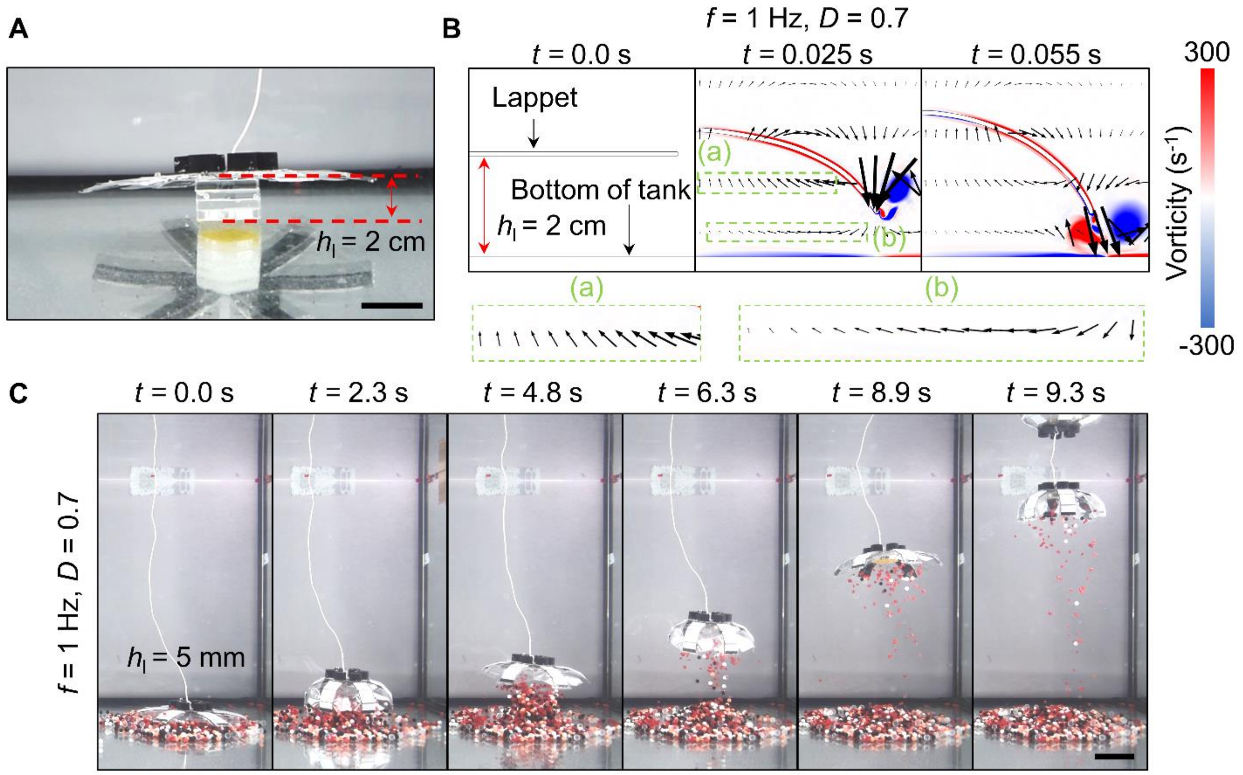

Figure S2. Effects of object sizes on contactless transportation. (**A**) A scheme showing the distance between the lappets and the bottom of the tank at the rest state before launching, h_l. In the current design, h_l was set to 2.0 cm to place the robot's center of mass as low as possible so that stability could be maintained during upward propulsion. (**B**) Simulation details on the drifted flow below the launched robot. In contrast to the upward velocity field, as is shown in the region closer to the lappet (a), the flow field closer to the bottom (b) tends to be horizontal with respect to the body. (**C**) Contactless transportation of objects smaller than 5 mm by decreasing h_l to 5 mm. Scale bar: 5 cm.

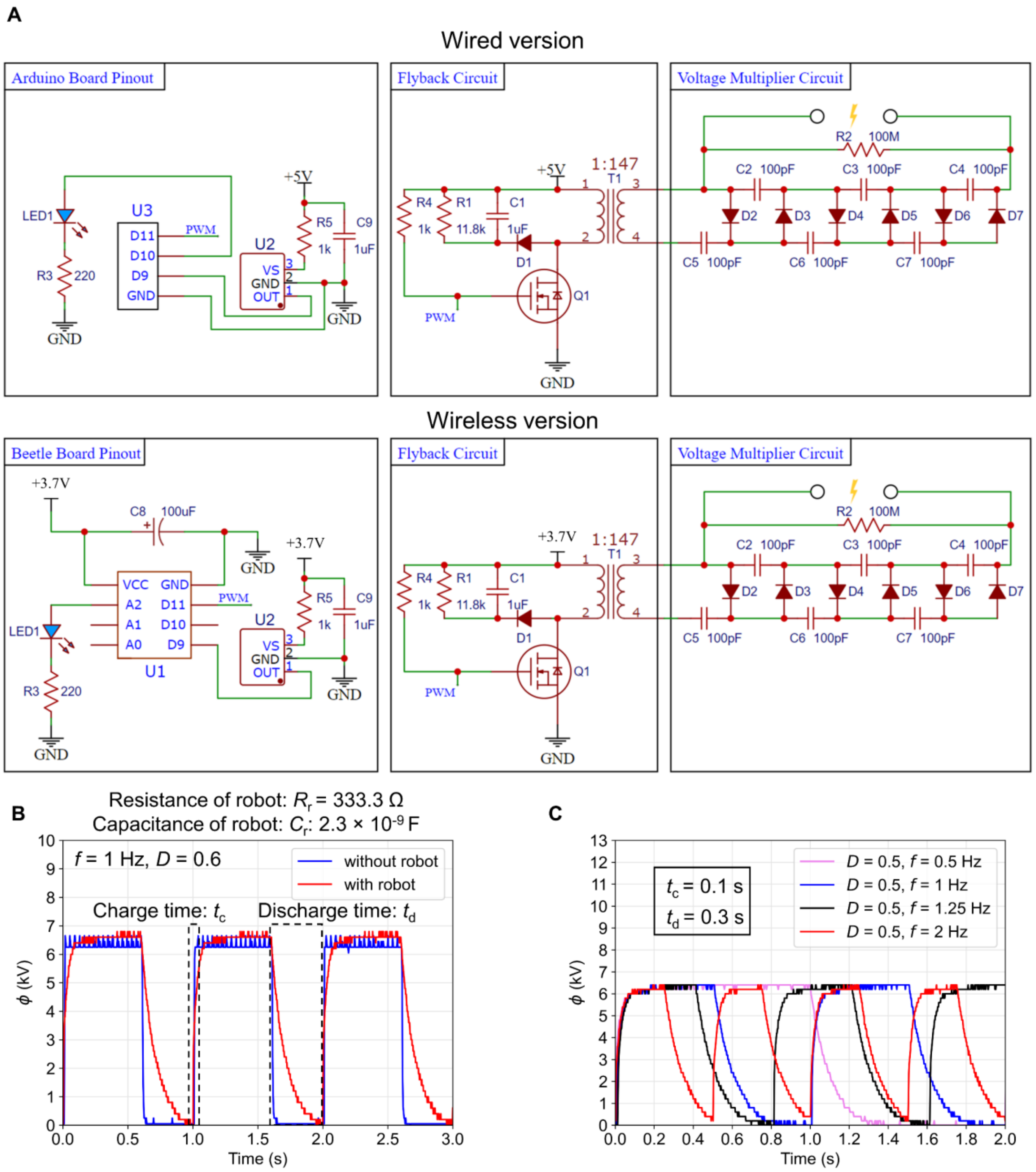

Figure S3. Electronics for high voltage generation and experimental characterization. (**A**) The wired and wireless versions of the electronics. The bills of materials are shown in **Table S2**. A high voltage was generated using the flyback stage and voltage multiplier stage. (**B**) The voltage applied to the HASEL jellyfish robot. In this example, the circuits without a connection to the robot output the square signal with the maximum voltage Φ_{max} = 6.5 kV, frequency f = 1 Hz, and duty cycle D = 0.6. Since the robot could be considered a serial connection between a resistor and

a capacitor, its integration into the circuits would influence the time responses. The quantified charge time t_c was around 0.1 s, and the discharge time t_d was around 0.3 s. (**D**) A confirmation of t_c and t_d by varying f. We decided the t_c and t_d based on the electronics and robot design, and they were not affected by the actuation parameters.

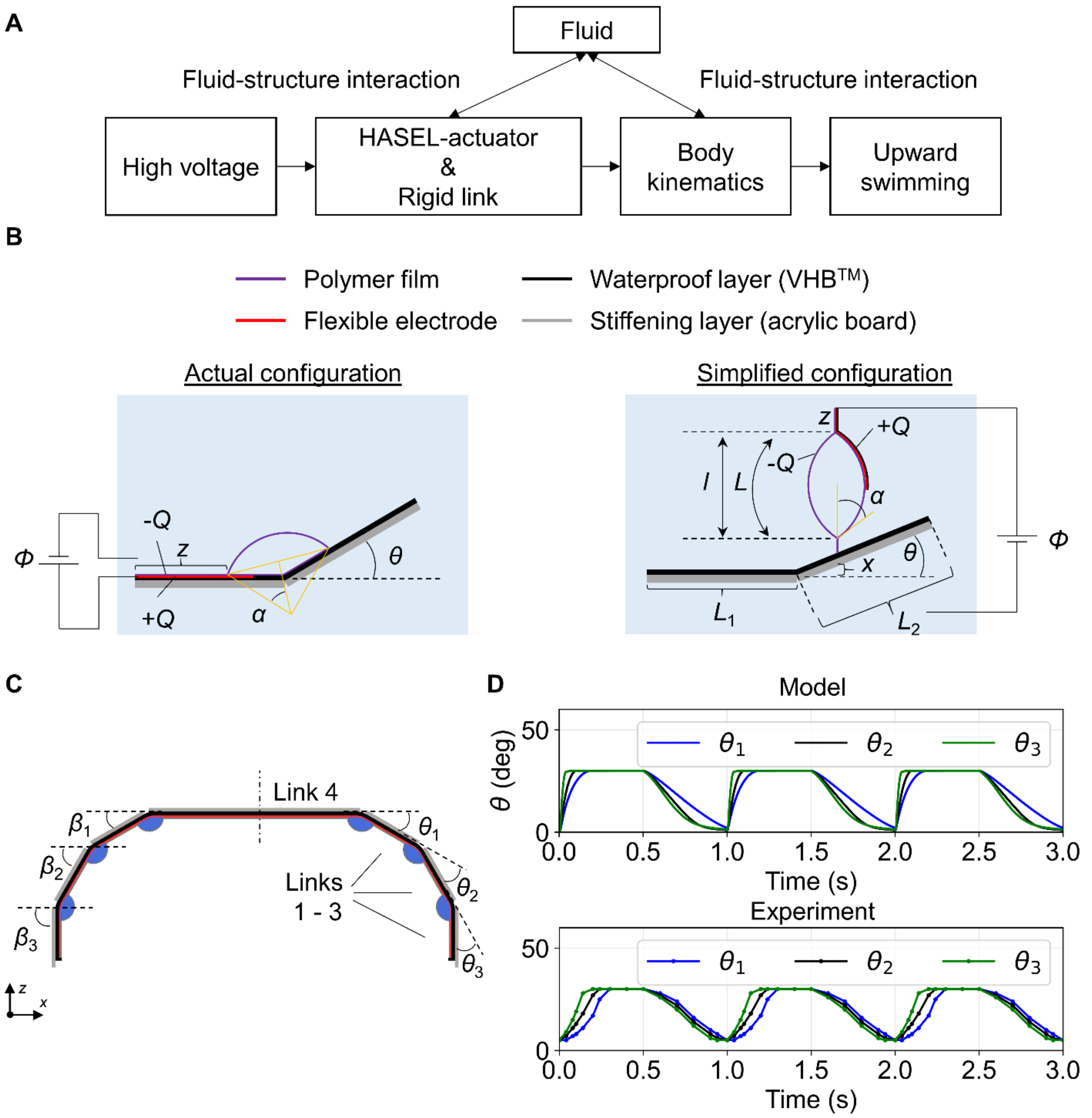

Figure S4. A model for the HASEL jellyfish robot's dynamics. (**A**) The principle of modeling. (**B**) The actual configuration and simplified configuration for one HASEL-actuated joint. Unlike the actual configuration, the relationship between z, θ, and the generalized coordinate α could be explicitly expressed in the simplified configuration for modeling purposes. (**C**) Generalized coordinates (θ_1, θ_2, θ_3) or (β_1, β_2, β_3) to describe the robot's body kinematics. (**D**) Modeling the experimental results of the body kinematics (f = 1 Hz, D = 0.5).

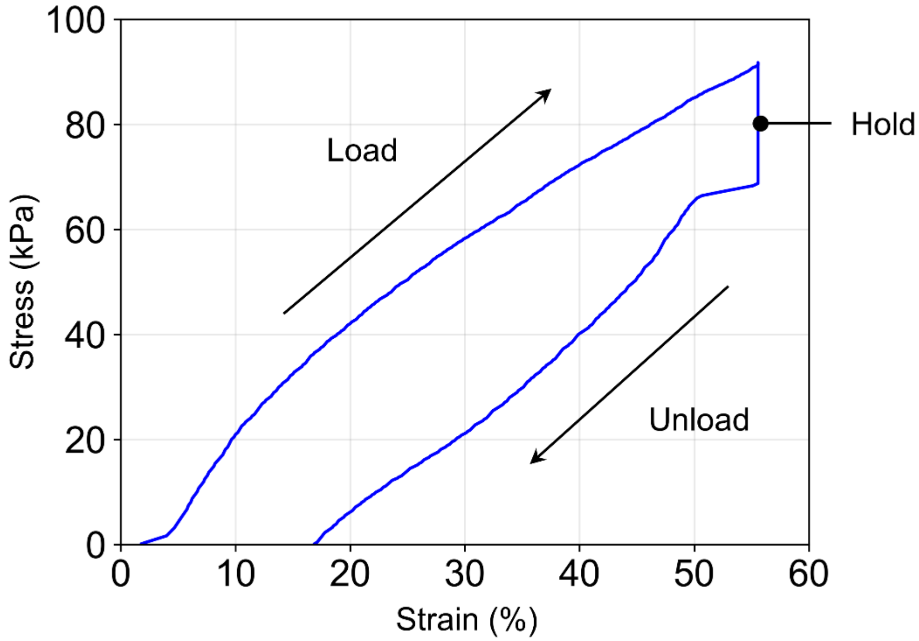

Figure S5. Elastic hysteresis of the viscoelastic waterproof layer made of VHB^{TM} 5925F tape. For the experimental characterization, we set the loading and unloading speed to 1 mm/s, the maximum elongation to 5 mm, and the holding time to 5 s.

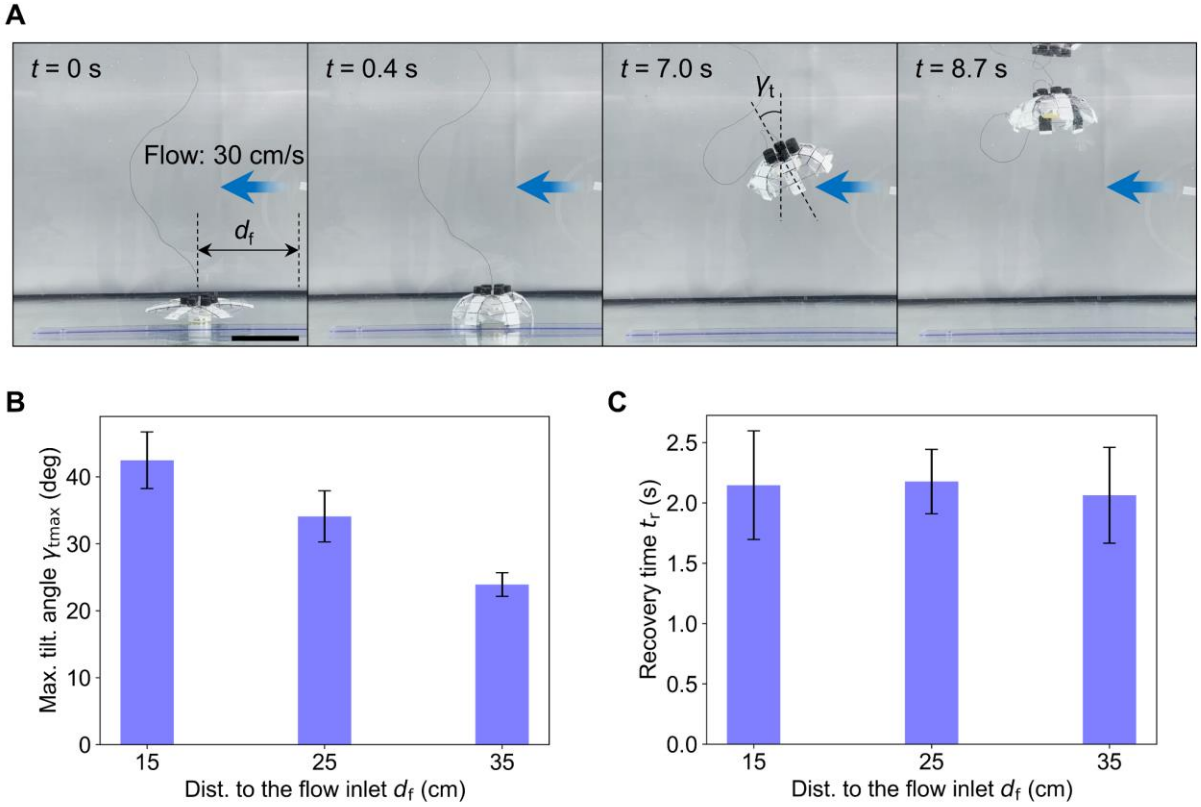

Figure S6. Effects of external flow on the upward propulsion. (**A**) Experimental snapshots of the upward propulsion under the flow. (**B**) Maximum achieved tilting angles γ_{tmax} at different locations away from the external flow inlet (p = 9.3 × 10^{-5}, one-way ANOVA test). (**C**) Recovery time t_r at different locations (p = 0.45, one-way ANOVA test). The actuation signal was set f = 1.25 Hz, D = 0.5. The error bars represent the SDs of the means for $n \geq 4$ experimental trials. Scale bar: 10 cm.

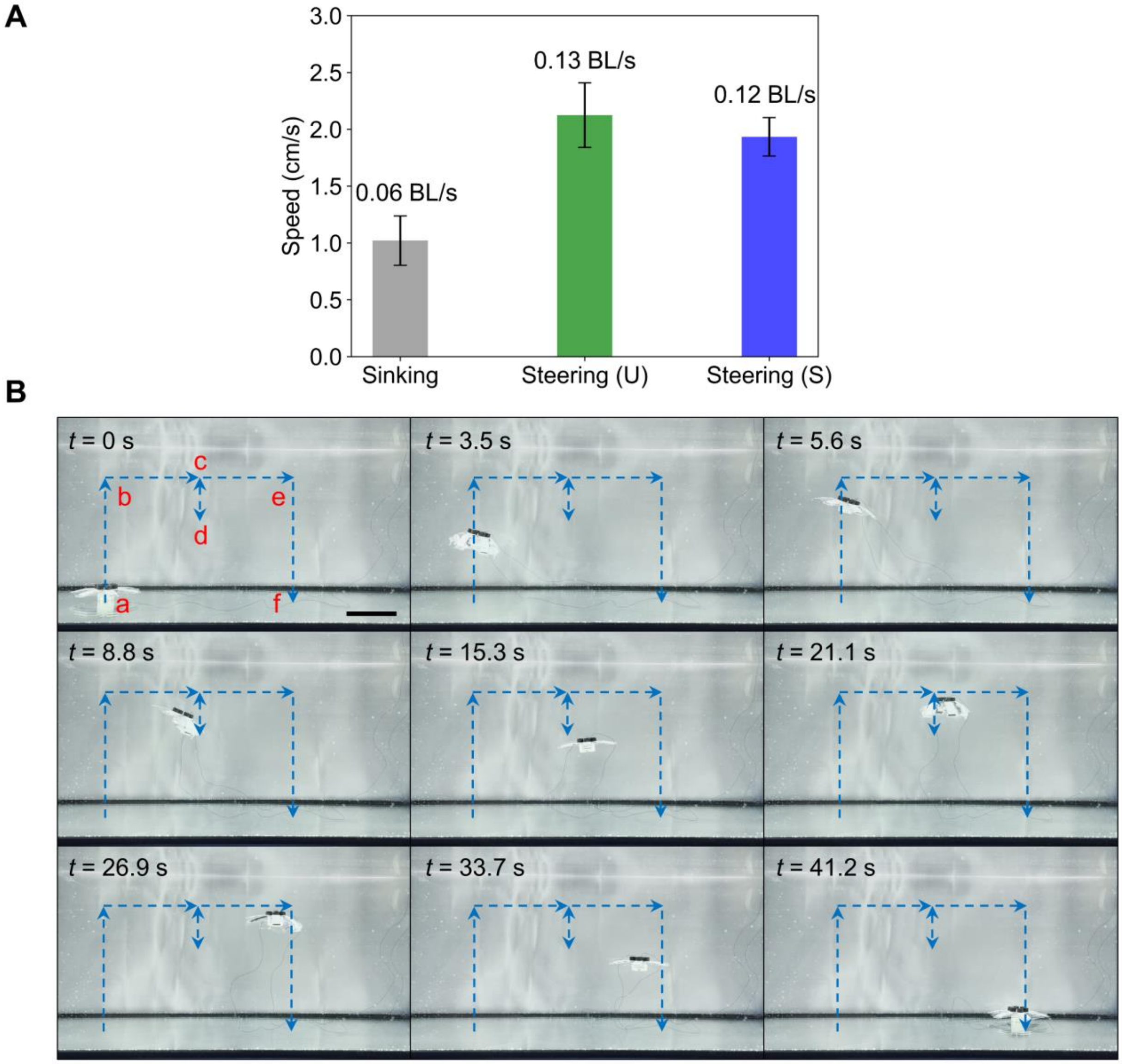

Figure S7. Investigations of steering capability. (**A**) Characterization of the speed during the sinking, steering while swimming upwards, and steering while sinking downwards. No significant difference was observed between the steering speeds (p = 0.28, one-way ANOVA test). 'U' refers to upward propulsion and 'S' refers to sinking. (**B**) Demonstration of M-shaped path following. The via points include a – f. The actuation signal was set f = 1.25 Hz, D = 0.5. The error bars represent the SDs of the means for $n \geq 5$ experimental trials. Scale bar: 10 cm.

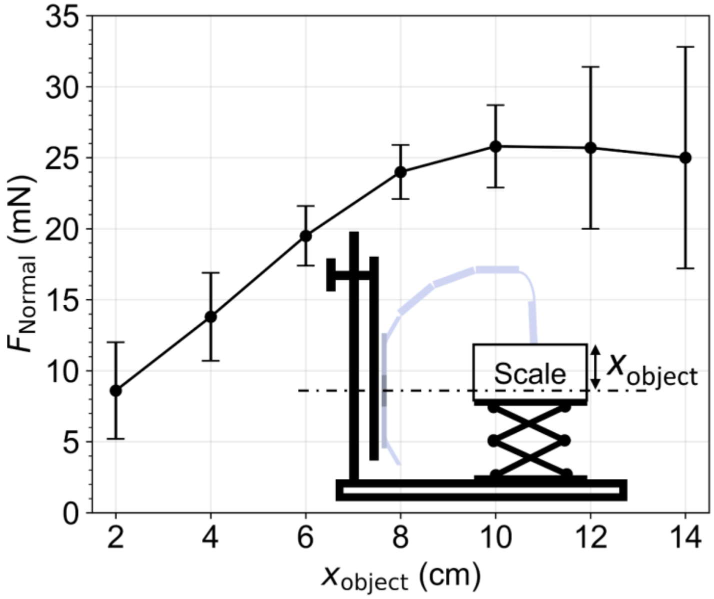

Figure S8. Relations between the size of object x_{object} and the grasping force F_{normal} (normal to the surface in contact). Here we used the lab scale with adjustable height to simulate the object with different sizes. The weight of the lappet has been deducted. The error bars represent the SDs of the means for $n \geq 5$ experimental trials.

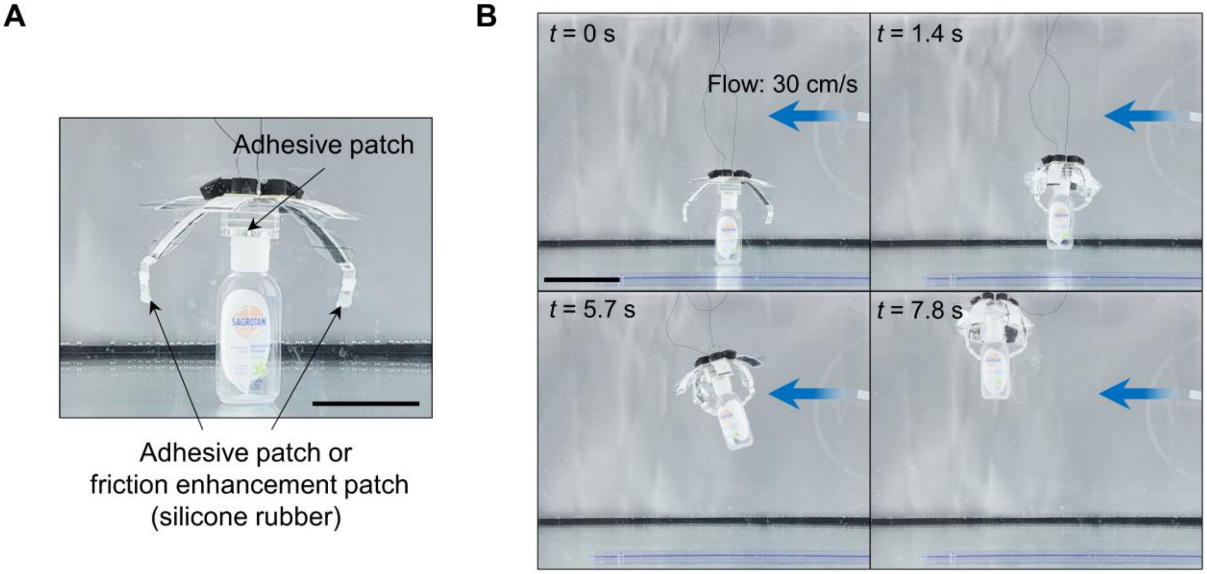

Figure S9. Object transportation under the external flow. (**A**) A feasible strategy to enhance the grasping and propulsion given the external flow. (**B**) Demonstration of the transportation under the flow. Scale bar: 10 cm.

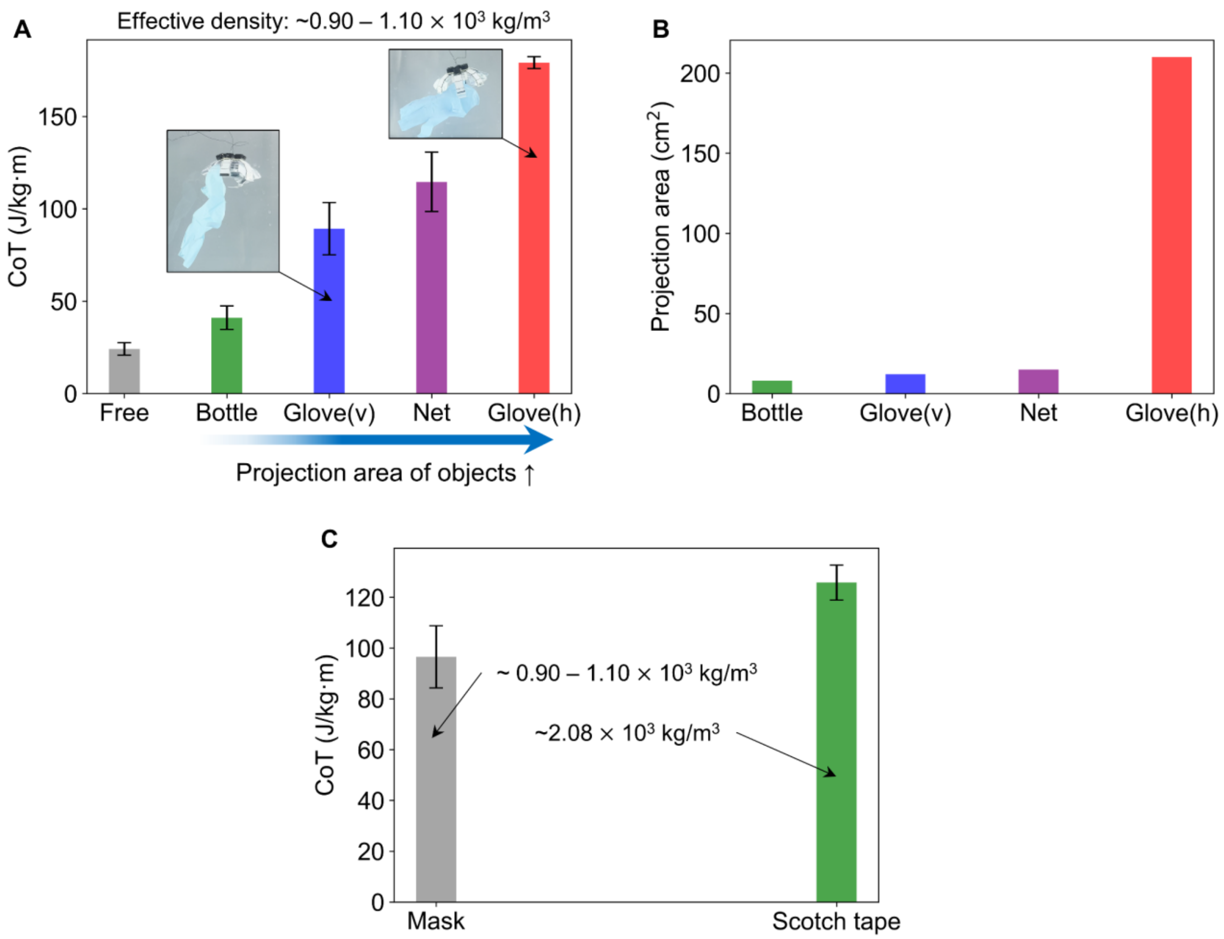

Figure S10. Effects of the transported objects on the CoT. (**A**) Comparison of CoT among different objects. ‘v’ is short-termed for ‘vertical’ and ‘h’ is for ‘horizontal’. (**B**) Estimation of the projection area facing the propulsion direction for different objects. (**C**) CoT of the transportation using two robots. The actuation signal was set f = 1.25 Hz, D = 0.5. The error bars represent the SDs of the means for $n \geq 3$ experimental trials.

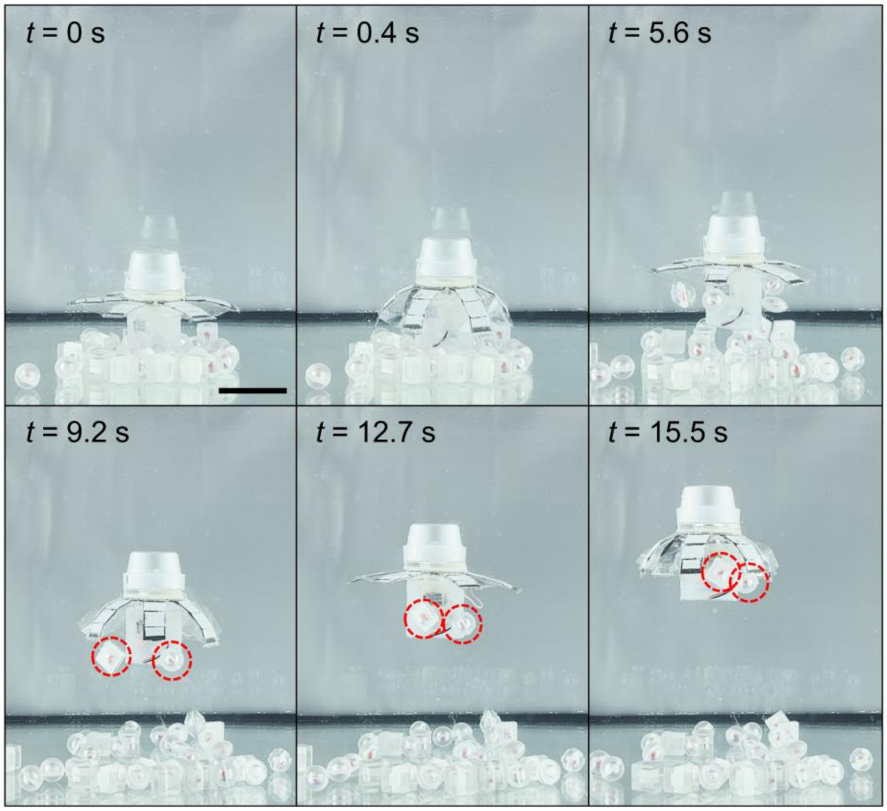

Figure S11. Demonstration of contactless manipulation by the wireless prototype. The actuation signal was set f = 1.25 Hz, D = 0.5. Scale bar: 5 cm.

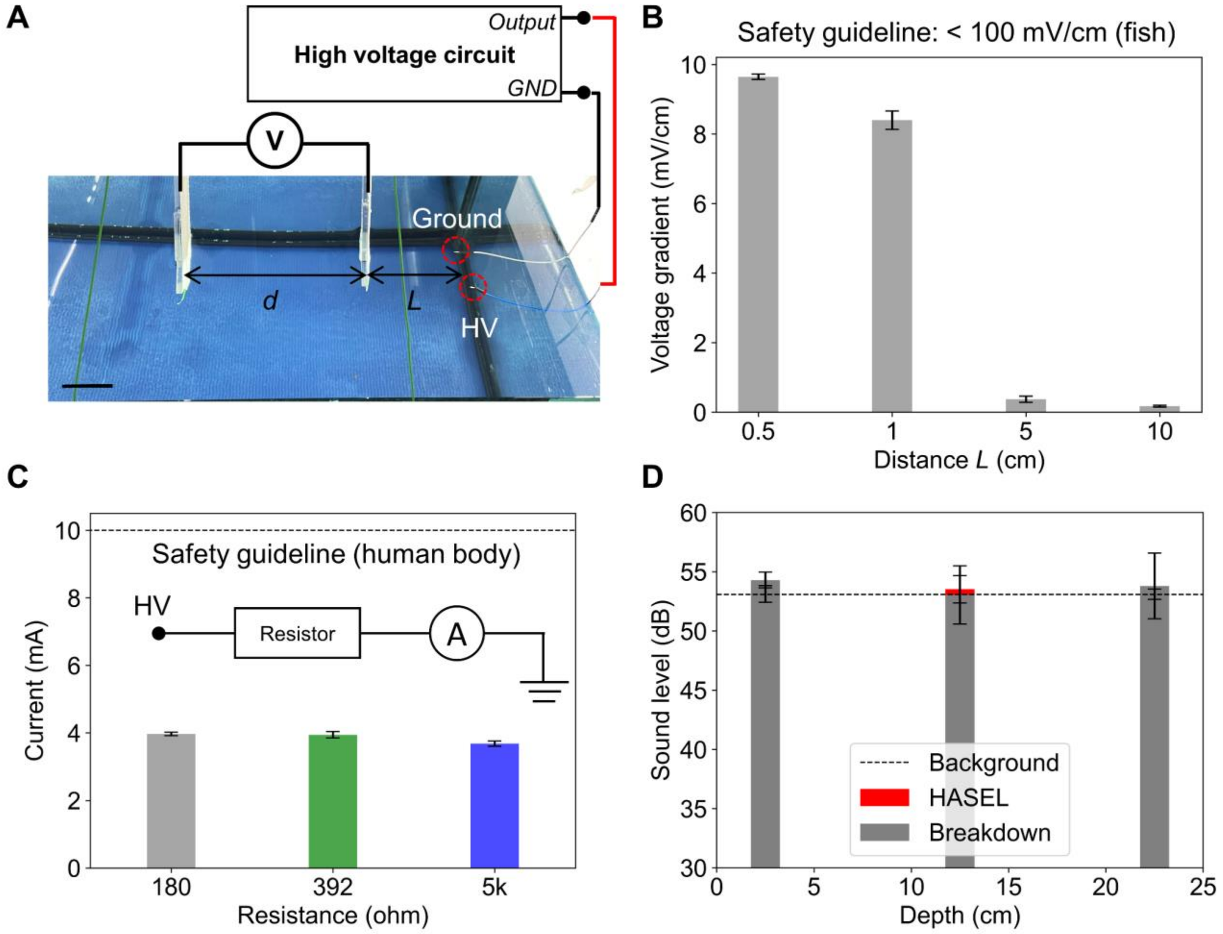

Figure S12. Investigations on electrical and acoustic safety. (**A**) Experimental setup to quantify the voltage gradient. Scale bar: 5 cm. (**B**) Relations between the voltage gradient and the distance to the HV cable. HV cable and ground cable were directly positioned in water with constant output. (**C**) Current measurements when different resistors were directly connected to the HV and ground cables in a serial configuration. (**D**) Sound levels of the background, normal operation, and breakdown.

Table S1. Values of various experimental parameters

Parameters	Value
The thickness of the Mylar film t_m (μm)	15.0
The thickness of the VHB™ tape t_v (mm)	0.6
The thickness of the link t_l (mm)	1.0
The gap between the links l_g (mm)	0.5 – 1
The width of the shell w (mm)	15.0
The width of the VHB™ tape w_v (mm)	19.0
The width of the link w_l (mm)	19.0
The Young's modulus of the VHB™ E_v (MPa)	0.45
The Poisson's ratio of the VHB™ v_v	0.499
The Young's modulus of the Mylar film E_m (GPa)	2
The relative permittivity of the Mylar film ε_m	3.2
The vacuum permittivity ε_0 (F/m)	8.9×10^{-12}
The length of the electrode h (mm)	9.0
The length of the electrode-free shell area r (mm)	6.0
The length of the shell L (mm)	$L = h + r = 15.0$
The volume of the injected silicone oil in each shell V_o (ml)	0.13
The density of the silicone oil (kg/m^3)	0.93×10^3
The kinematic viscosity of the silicone oil (cSt)	5
The estimated resistor of the robot R_r (Ω)	333.3
The estimated capacitance of the robot C_r at the fully actuated state (F)	2.3×10^{-9}
The mass of the robot m_r (g)	51.9 ± 0.3
The effective density of the robot in water ρ_r (kg/m^3)	1.23×10^3

Table S2. Bills of materials for the wired and wireless versions of the electronics

Qty	Parts	Description	Device part number
1	U1	DFRobot Beetle board	DFR0282
2	U2	IR receiver	TSOP38238
1	U3	Arduino Uno board	A000066
2	Q1	N-channel MOSFET	DMN3060LWQ-7
2	T1	Transformer	TW-G-DHXQ-211
2	R1	11.8 kΩ resistor	RC1206FR-0711K8L
10	R2	20 MΩ resistor	VR37000002005JR500
2	R3	220 Ω resistor	CRGH1206J220R
4	R4, R5	1 kΩ resistor	CRGH1206F1K0
4	C1, C9	1 µF capacitor	12061C105KAT2A
12	C2, C3, C4, C5, C6, C7	100 pF capacitor	C1812C101JHGACTU
1	C8	100 µF capacitor	ECA1VM101
2	D1	Fast rectifier diode	FR107G R0
12	D2, D3, D4, D5, D6, D7	HV diode	TC-HV5
2	LED1	Blue LED	150120BS75000
1	+3.7V	Battery with 3.7V/350 mAh	LP552035

Table S3. External power and speed of various jellyfish robots

Robot	Wired or wireless	Mass (kg)	Max. Dimension (m)	Speed (m/s)	Speed (BL/s)	External power (W)	CoT (J/kg·m)
Jennifish (*15*)	***Wireless***	0.38	0.21	0.03	0.14	2.29	200.88
RoboJelly (*19*)	Wired	0.24	0.16	0.03	0.19	17	2266.06
DEA-jellyfish-wired (*20*)	Wired	0.06	0.16	0.002	0.01	0.25*	2525.25
DEA-jellyfish-wireless (*20*)	***Wireless***	0.23	0.16	0.003	0.02	0.25*	339.67
KryptoJelly (*17*)	Wired	0.65	0.21	0.002	0.01	360	307692.31
Synthetic jellyfish (*18*)	Wired	0.04	0.15	0.02	0.13	110	137500
Cyro (*14*)	***Wireless***	76	1.7	0.09	0.05	70	10.87
**HASEL jellyfish-wired#**	**Wired**	**0.05**	**0.16**	**0.05**	**0.31**	**0.06**	**24.14**
**HASEL jellyfish-wireless#**	***Wireless***	**0.17**	**0.16**	**0.02**	**0.12**	**0.05**	**15.88**

* The values are based on the maximum output power of the high voltage direct current (HVDC) converters.

# These values were calculated based on f = 1.25 Hz, D = 0.5.

Table S4. Comparison with millimeter jellyfish robot about contactless manipulation (*13*)

Actuation method	Magnetic	Electrohydraulic
Max. dimension (mm)	6.00	160.00
Max. number of transported objects	6	8
Diameter of the object (mm)	0.55	20.00
Size of the object w.r.t. dimension	0.09	0.13
Max. transported distance (mm)	18.00	450.00
Transported distance w.r.t. dimension	3.00	2.81

Table S5. Properties of the transported objects

Objects	Mass (g)	Effective density (g/cm^3)	Projection area (cm^2)
Plastic bottle	71.3	1.10	8.0
Rubber glove	10.7	1.10	12.0 (vertical); 210.0 (horizontal)
Net	6.1	0.90	15.0
Mask	19.0	0.90	126.0
Scotch tape	6.8	2.08	8.0

Table S6. A comparison between the wired and wireless HASEL jellyfish robots*

Version	Wired	Wireless
Weight (kg)	0.05	0.17
Estimated input power (W)	0.06	0.05
Effective density (kg/m^3)	1.23×10^3	1.23×10^3
Max. actuation HV (kV)	6.5	6.2
Average speed (cm/s)	5.0	2.0
Re	8493.7	3327.9
CoT (J/ kg·m)	24.14	15.88

* The comparison was evaluated at f = 1.25 Hz, D = 0.5; the average values are reported here.

Supplementary Movies

Movie S1. Propulsion performance and flow visualization by fluorescein dye

Movie S2. Contactless object manipulation

Movie S3. Steering by actuating individual lappets

Movie S4. Propulsion with grasped objects

Movie S5. Contactless object manipulation by three HASEL jellyfish robots

Movie S6. Propulsion with a grasped object by two HASEL jellyfish robots

Movie S7. Fluidic mixing by three HASEL jellyfish robots

Movie S8. A wireless prototype
